# Anti-infective potential of hydroalcoholic extract of *Punica* *granatum* peel against gram-negative bacterial pathogens

**DOI:** 10.12688/f1000research.17430.2

**Published:** 2019-04-08

**Authors:** Chinmayi Joshi, Pooja Patel, Vijay Kothari

**Affiliations:** 1 Institute of Science, Nirma University, Ahmedabad, Gujarat, 382481, India

**Keywords:** Punica granatum peel, Quorum Sensing, Post Extract Effect, Prebiotic, Microwave Assisted Extraction

## Abstract

**Background:** *Punica granatum* extracts have been prescribed in traditional medicine for management of a variety of disease conditions including microbial infections. Generation of scientific evidence for validation of *P. granatum* peel extract’s anti-pathogenic efficacy is required.

**Methods: **Hydroalcoholic extract of *P. granatum* peel (PGPE), prepared by microwave assisted extraction method was evaluated for its quorum-modulatory potential against two different human-pathogenic bacteria viz. *Chromobacterium violaceum* and *Pseudomonas aeruginosa*.

**Results: **This extract was able to modulate *in vitro* production of quorum sensing-regulated pigments in both these test bacteria at ≥5 μg/ml. Virulence traits of *P. aeruginosa* like haemolytic activity, and biofilm formation were negatively affected by the test extract, and it also made *P. aeruginosa* more susceptible to lysis by human serum. Antibiotic susceptibility of both test bacteria was modulated owing to pre-treatment with PGPE. Exposure of these test pathogens to PGPE (≥0.5 μg/ml) effectively reduced their virulence towards the nematode *Caenorhabditis elegans*. Repeated subculturing of *P. aeruginosa* on PGPE-supplemented growth medium did not induce resistance to PGPE in this notorious pathogen, and this extract was also found to exert a post-extract effect on *P. aeruginosa. *Individual constituent phytocompounds of PGPE were found to be less efficacious than the whole extract. PGPE seemed to interfere with the *signal-response* machinery of *P. aeruginosa* and *C. violaceum*. PGPE also exhibited notable prebiotic potential by promoting growth of probiotic strains- *Bifidobacterium bifidum* and *Lactobacillus plantarum *at ≤50 μg/ml.

**Conclusions: **This study indicates PGPE to be an effective antipathogenic and prebiotic preparation, and validates its therapeutic use mentioned in traditional medicine. This study also emphasizes the need for testing any bioactive extract at broadest possible concentration range, particularly *in vivo*, so that an accurate picture of dose-response relationship can emerge.

## Introduction

Antimicrobial resistance (AMR) has been recognized as a global problem. Globalization and international travel has increased the vulnerability of any country to infections prevalent in other countries. Rapid spread of resistant factors across different species of pathogenic bacteria, particularly through horizontal gene transfer permitting promiscuous exchange of genetic material, presents a global threat to public health. It is estimated that every 5 minutes one child on the earth dies because of bacterial resistance to antibiotics ( ESRC Report, 2014). One of the most problematic features of AMR is that resistance to a new antimicrobial can begin simultaneous to its development, meaning that simply developing new bactericidal antibiotics is not the panacea.

Hitherto, antimicrobial therapy has largely remained dependent on conventional bactericidal antibiotics. However, owing to their killing effect, such antibiotics pose considerably strong selection pressure on the pathogen populations to evolve resistant phenotypes. This necessitates thinking of alternative approaches for handling microbial infections, which asks for identifying novel targets, other than those (e.g. cell wall synthesis, nucleic acid/protein synthesis, cell membrane) targeted by current antibiotics ( Beceiro *et al.,* 2013; Fair & Tor, 2014; Kohanski *et al.,* 2010). Among such possible novel targets, quorum sensing (QS) has attracted considerable attention from the researchers working in the area of AMR. QS is a mechanism of chemical signalling (via acyl homoserine lactones (AHL) in gram-negative bacteria, and autoinducing peptides (AIP) in gram-positive bacteria) based process, which allows the bacterial population to modulate its behaviour in relation to its cell density ( Moore *et al.,* 2015). Though a large part of the bacterial genome (including the genes coding for virulence) is regulated by QS, it is not that essential for their survival as an individual cell, hence quorum-modulatory agents can be expected to exert their anti-infective/antipathogenic effect on susceptible bacterial populations, without necessarily putting a strong selection pressure on them. 

Though the modern medicine has almost always focused on search of purified active molecules as therapeutic leads, knowledge of Indian/Chinese/Arabian traditional medicine makes us aware of a variety of plant extracts and inorganic formulations prescribed for management of infections. One such widely mentioned item in these ancient systems of complementary and alternative medicine, including the Indian system *Ayurved*, is *Punica granatum* L. extracts. This plant belongs to the family Lythraceae (Punicaceae), and the common English name is pomegranate. Different parts of this plant have been applied in traditional medicine to treat a variety of health problems. A peep into literature with particular focus on its peel reveals that in Egyptian culture, common ailments such as inflammation, diarrhea, intestinal worms, cough, and infertility were treated by exploiting pomegranate peel extract ( Ismail *et al.,* 2012). *P. granatum* has been indicated in traditional Indian and Iranian medicine for its antimicrobial potential, for treatment of throat infections (e.g. sore throat caused by bacterial infection), diarrhea, wound healing, etc. ( Hosein Farzaei *et al.,* 2014). In *Charak Samhita (Sutrsthana)* pomegranate has been mentioned as an important ingredient of a *Yavagu* formulation for treatment of dysentery. In India, Tunisia, and Guatemala, decoction of dried peels of *P. granatum* is employed externally as well as internally as an astringent and germicide, and utilized for treating aphthae and diarrhoea. In *Ayurvedic* medicine, this plant described as ‘ *dadim’* (its Sanskrit name), is considered as a ‘blood purifier’ and suggested to cure parasitic infections ( Colombo *et al.,* 2013). A search in the IMPPAT database ( https://cb.imsc.res.in/imppat/basicsearch/therapeutics) using the search term “ *Punica granatum*” yields 75 different therapeutic uses, of which multiple conditions (vaginal trichomoniasis, pharyngitis, periodontitis, paracoccodioidomycosis, malaria, leshminasis, Klebseiella Pneumonia, *Helicobacter pylori* infection, gonorrhea, gingivitis, genital herpes, dysentery, diarrhea, conjunctivitis, cholera, etc.) for which *P. granatum* is indicated can involve microbial infections. Ethnobotanical use of *P. granatum* for treatment of diarrhoea has been documented from South Africa too ( Mathabe *et al.,* 2006). Mexican traditional medicine also mentions utility of *P. granatum* in treatment of gastrointestinal disorders ( Alanis *et al.,* 2005). Based on Iranian traditional medicinal practice, Hajimahmoodi *et al.* 2011 demonstrated peel extracts of eight cultivars of pomegranate to be effective against *Helicobacter pylori*. One ethnomedical practice reported from the Paliyar tribe from Tamilnadu in India involves taking internally, dried fruit coat of *P. granatum* after grinding and mixing with water, to treat stomach ache and diarrhoea ( Duraipandiyan *et al.,* 2006). Ayurvedic pharmacopeia of India mentions formulations containing rind of *P. granatum* to be useful in conditions such as fever, dysentery, bacteremia, and infections of oral cavity ( Khare, 2004). Decoction containing *P. granatum* peel is used for gastrointestinal benefit in Algeria too ( Gharzouli *et al.,* 1999). Despite their wide use in medicinal texts of various cultures, efficacy of pomegranate extracts needs to be validated through appropriate experiments for their wider acceptance in the modern world.

This study aimed at investigating the possible anti-infective potential of pomegranate peel extract against two gram-negative bacteria, *Pseudomonas aeruginosa* and *Chromobacterium violaceum*. *P. aeruginosa* is amongst the most notorious bacterial pathogens, associated with chronic and acute urinary and respiratory tract infections, and its carbapenem-resistant phenotype has been listed as a pathogen of ‘critical’ priority against which new antimicrobials are urgently warranted ( WHO report, 2017). *C. violaceum* is widely used in QS studies and is also being viewed as an emerging pathogen ( Kothari *et al.,* 2017). WHO advises for future research to focus on the development of new antibiotics specifically against drug-resistant gram-negative bacteria ( WHO report, 2017). In both gram-negative bacteria selected in this study, pigment production is a trait, which is reported to be under control (largely but not fully) of their QS machinery, and this was used as a marker trait by us while assaying effect of our extract on these bacteria. QS-regulated pigments produced by these bacteria are: pyoverdine and pyocyanin in *P. aeruginosa* ( Adonizio *et al.,* 2007; Lee & Zhang, 2015), and violacein in *C. violaceum* ( Vasavi *et al.,* 2013).

## Methods

### Materials

All the growth media/ media ingredients/ assay reagents, and antibiotics were procured from Himedia, unless specified otherwise. TLC plates, and all organic solvents used in this study were procured from Merck. Whatman paper, and bacteriological filters were from Axiva (Haryana). Catechin was from Sigma Aldrich (USA). Triton X-100 was from CDH (New Delhi). All experiments were performed with the same set of reagents.

### Test organisms

*C. violaceum* (MTCC 2656), and *Lactobacillus plantarum* (MTCC 2621), were procured from MTCC (Microbial Type Culture Collection, Chandigarh), whereas *Bifidobacterium bifidum* (NCDC 255) was procured from NCDC (National Collection of Dairy Cultures), NDRI (National Dairy Research Institute, Karnal). *C. violaceum* was grown in nutrient broth (HiMedia, Mumbai), *L. plantarum* was grown in Lactobacillus MRS medium (HiMedia, Mumbai), and *B. bifidum* was grown on MRS with 0.05% cysteine. Incubation temperature and time for *C. violaceum*, *L. plantarum*, and *B. bifidum* was 37°C, and 22–24 h. Antibiotic susceptibility profile of the bacterial strains used in this study was generated using the antibiotic discs-Dodeca Universal – I, Dodeca G - III – Plus, and Icosa Universal -2 (HiMedia, Mumbai). *C. violaceum* was found to be resistant to cefadroxil (30 µg), ampicillin (10 µg), cloxacillin (1 µg), and penicillin (10 µg). Culture of *P. aeruginosa* was obtained from Microbiology Department, M.G. Science Institute, Ahmedabad. Pseudomonas agar (HiMedia, Mumbai) was used for the maintenance of the culture. Strain of *P. aeruginosa* was found to be resistant to amoxicillin (30 µg), cefadroxil (30 µg), ampicillin (10 µg), cloxacillin (1 µg), penicillin (10 µg), chloramphenicol (30 µg), cefixime (5 µg), clindamycin (2 µg), and nitrofurantoin (300 µg).

### Plant material

Peels of *P. granatum* were procured from the fruits purchased from local market in the city of Ahmedabad. Plant material (ref no: GU/Bot/29/4/2015) was authenticated for its unambiguous identity by Dr Archana Mankad (Deparment of Botany, Gujarat University, Ahmedabad). The plant name was checked against http://www.theplantlist.org on April 20, 2018. Collected peels were shade-dried, before being used for extract preparation. We also analysed another pomegranate fruit extract as ‘Pomella’ by Pharmanza Herbal Pvt. Ltd., which contained not less than 30% punicalagin.

### Extract preparation

A hydroalcoholic extract of the plant material was prepared in 50% ethanol using the microwave-assisted extraction (MAE) method ( Kothari *et al.,* 2009). Dry peel powder (total 5 g; 1 g in each vessel) was soaked into the solvent in a ratio of 1:50, and subjected to microwave heating (Electrolux EM30EC90SS) at 720 W. Total heating time was 120 seconds, with intermittent cooling. This was followed by centrifugation (at 10,000 rpm for 15 min.), and filtration with Whatman paper #1 (Axiva, Haryana). Solvent was evaporated from the filtered extract and then the dried extract was reconstituted in dimethyl sulfoxide [DMSO (absolute); Merck] for broth dilution assay. Reconstituted extract was stored under refrigeration for further use. Extraction efficiency was calculated as percentage weight of the starting dried plant material, and its value was 42% w/w. 

### Broth dilution assay

Assessment of QS-regulated pigment production by test pathogens in presence or absence of the test formulation, was done using broth dilution assay ( Patel *et al.,* 2017). Organisms were challenged with different concentrations (0.5-500 μg/ml) of *P. granatum* peel extract (PGPE). Nutrient broth or Pseudomonas broth (peptic digest of animal tissue 20 g/l, potassium sulphate 10 g/l, magnesium chloride 1.4 g/l, pH 7.0 ± 0.2) was used as a growth medium. Inoculum standardized to 0.5 McFarland turbidity standard was added at 10% v/v, to the media supplemented with required concentration of PGPE, followed by incubation at appropriate temp for each organism. Appropriate vehicle control containing DMSO was also included in the experiment, along with abiotic control (containing extract and growth medium, but no inoculum). Catechin (50 μg/ml; Sigma Aldrich, USA) was used as positive control, since it is already known QS inhibitor ( Nazzaro *et al.,* 2013; Vandeputte *et al.,* 2010).

### Measurement of bacterial growth and pigment production

At the end of the incubation, bacterial growth was quantified at 764 nm ( Joshi *et al.,* 2016). This was followed by pigment extraction and quantification, as per the method described below for each of the pigments. Purity of each of the extracted pigment was confirmed by running a UV-vis scan (Agilent Cary 60 UV-visible spectrophotometer). Appearance of single major peak (at the λ _max _reported in literature) was taken as indication of purity.

### Violacein extraction

Extraction of violacein from *C. violaceum* culture was executed as described by Choo *et al.* (2006). A total of 1 ml of the culture broth was centrifuged (Eppendorf 5417 R) at 15,300 *g* for 10 min at room temperature, and the resulting supernatant was discarded. The remaining cell pellet was resuspended into 1 ml of DMSO, and vortexed, followed by centrifugation at 15,300 *g* for 10 min. The purple coloured violacein was extracted in the supernatant; OD was measured at 585 nm. Violacein unit was calculated as OD _585_/OD _764_. This parameter was calculated to nullify the effect of any change in cell density on pigment production.

### Pyoverdine and pyocyanin extraction

Extraction of pyoverdine and pyocyanin from *P. aeruginosa* culture was achieved as described in El-Fouly *et al.* (2015) and Unni *et al.* (2014), respectively. A total of 1 ml of the culture broth was mixed with chloroform (Merck, Mumbai) in 2:1 proportion followed by centrifugation at 12,000 rpm (13,520 *g*; REMI CPR-24 Plus) for 10 min. This resulted in formation of two immiscible layers. OD of the upper water-soluble phase containing yellow-green fluorescent pigment pyoverdine was measured at 405 nm. Pyoverdine Unit was calculated as OD _405_/OD _764_.

The lower chloroform layer containing pyocyanin was mixed with 0.1 N HCl (Merck; at the rate of 20%v/v), resulting in a colour change from blue to pink. Absorbance of this pyocyanin in acidic form was measured at 520 nm. Pyocyanin Unit was calculated as OD _520_/OD _764_.

### N- acyl-homoserine lactone (AHL) augmentation assay

AHL extraction: AHL was extracted from the bacterial culture broth as described by Chang *et al.* (2014). OD of overnight grown bacterial culture was standardized to 1.00 at 764 nm. It was centrifuged at 5000 *g* for 5 min. Cell free supernatant was filter sterilized using 0.45 µm filter (Axiva, Haryana), and was mixed with equal volume of acidified ethyl acetate [0.1% formic acid (Merck) in Ethyl acetate (Merck)]. Ethyl acetate layer was collected, and evaporated at 55°C, followed by reconstitution of the dried crystals in 100 µl phosphate buffer saline (pH 6.8). Identity of thus extracted AHL was confirmed by thin-layer chromatography (TLC). R _f _value of purified AHL from *C. violaceum* culture, while performing TLC [Methanol (60): Water (40); TLC Silica gel 60 F _254 _plates; Merck] McClean *et al.,* (1997) was found to be 0.70, near to that (0.68) reported for N-hexonyl homoserine lactone (C6-HSL) ( Shaw *et al.,* 1997). TLC of AHL extracted from *P. aeruginosa* resulted in three spots corresponding to R _f_ values of 0.43, 0.68, and 0.92 near to those (0.41, 0.68, 0.84) for C8-HSL, C6-HSL, and C4-HSL respectively.

Bacterial culture growing in presence of test formulation was supplemented with 2%v/v AHL after 6 hours of incubation, and at the end of a total 24-hour incubation pigments were extracted from AHL-supplemented experimental tubes, as well as AHL-non-supplemented control tubes. If the QS-regulated pigment production is found inhibited in both these tubes in comparison to bacteria growing in absence of extract as well as AHL, then the effect of test extract was interpreted as signal-response inhibitor, because if the test formulation would have acted as a signal-supply inhibitor, then exogenous supply of AHL should restore pigment formation by the bacteria.

### Hemolysis assay

This assay was done as described by Neun *et al.* (2015). OD _764_ of overnight grown culture was standardized to 1.00. Cell free supernatant was prepared by centrifugation at 15,300 g for 10 min. A total of 10 µl human blood (collected in heparinized vial) was incubated with this cell free supernatant for 2 h at 37°C, followed by centrifugation at 800 g for 15 min; 1% Triton X-100 (CDH, New Delhi) was used as a positive control. Phosphate buffer saline was used as a negative control. OD of the supernatant was read at 540 nm, to quantify the amount of haemoglobin released.

### Assay of bacterial susceptibility to lysis in presence of human serum

This assay was performed as described by Ferro *et al.* (2016). Serum was separated by centrifuging blood at 1,500 rpm (800 *g*) for 10 min. Bacterial culture grown in media with and without PGPE was centrifuged, and the cell pellet was reconstituted in PBS, so that the resulting suspension attains OD _764_=1. A total of 200 µl of this bacterial suspension from control or experimental tubes was mixed with 740 µl of PBS and 60 µl of serum. After incubation for 24 h at 37°C, absorbance was read at 764 nm. DMSO -treated cells (0.5%v/v) suspended in PBS served as control, against which OD of the PGPE-treated cells (serum-exposed) was compared. Tubes containing bacterial cells exposed neither to DMSO nor serum were also included in the experimental set-up, to nullify any interference from autolysis.

Human blood was obtained from the authors, who each gave their written informed consent. The use of this blood was approved by the Institutional Ethics Committee of the Institute of Science, Nirma University (approval no: EC/NU/18/IS/4).

### Catalase assay

The OD _764_ of the culture was adjusted to 1.00. Next, 400 µl of phosphate buffer was added into a 2 ml vial followed by 400 µl H _2_O _2. _To this 200 µl of the bacterial culture was added, and the mixture was incubated for 10 min. Then 10 µM of sodium azide was added to stop the reaction ( Iwase *et al.,* 2013), followed by centrifugation at 12,000 rpm for 10 min. OD of the supernatant was measured at 240 nm to quantify remaining H _2_O _2_ ( Weydert & Cullen, 2010), with phosphate buffer as blank. Disappearance of the substrate H _2_O _2 _was taken as the measure of the catalase activity.

### Assay for biofilm formation, eradication and viability

In this assay, the control and experimental groups contained nine test tubes. In each group, three subgroups were made. First subgroup of three test tubes in the experimental group contained Pseudomonas broth supplemented with PGPE, whereas remaining six tubes contained Pseudomonas broth with no PGPE on first day of experiment. All these tubes were inoculated with inoculum (10%v/v) standardized to 0.5 McFarland turbidity standard (making the total volume in the tube 1 ml), followed by incubation at 37°C for 24 h under static condition, which resulted in formation of biofilm as a ring on walls of the glass tubes. This biofilm was quantified by crystal violet assay ( Patel *et al.,* 2013), preceded by quantification of bacterial cell density and pigment. Content from the remaining six test tubes from rest of the two subgroups were discarded following cell density and pigment estimation, and then the biofilms remaining on inner surface of these tubes were washed with phosphate buffer saline (PBS; pH 7) to remove loosely attached cells. Now, 2 ml of minimal media (Sucrose 15 g/l, K _2_HPO _4_ 5.0 g/l, NH _4_Cl 2 g/l, NaCl 1 g/l, MgSO _4_ 0.1 g/l, yeast extract 0.1 g/l, pH 7.4±0.2) containing PGPE, was added into each of these tubes, so as to cover the biofilm completely, and tubes incubated for 24 h at 37°C. At the end of incubation, one subgroup of 3 tubes was subjected to crystal violet assay to know whether any eradication of the pre-formed biofilm has occurred under the influence of PGPE, and the last subgroup of 3 tubes was subjected to viability assessment through MTT assay. For the crystal violet assay, the biofilm- containing tubes (after discarding the inside liquid) were washed with PBS in order to remove all non-adherent (planktonic) bacteria, and air-dried for 15 min. Then, each of the washed tubes was stained with 1.5 ml of 0.4% aqueous crystal violet solution for 30 min. Afterwards, each tube was washed twice with 2 ml sterile distilled water and immediately de-stained with 1500 µl of 95% ethanol. After 45 min of de-staining, 1 ml of de-staining solution was transferred into separate tubes, and read at 580 nm. For the MTT assay ( Trafny *et al.,* 2013), the biofilm-containing tubes (after discarding the inside liquid) were washed with PBS in order to remove all non-adherent (planktonic) bacteria, and air-dried for 15 min. Then 900 µl of minimal media was added into each tube, followed by addition of 100 µl of 0.3% MTT [3-(4,5-Dimethylthiazol-2-yl)-2,5-Diphenyltetrazolium Bromide, HiMedia]. After 2 h incubation at 37°C, resulting purple formazan derivatives were dissolved in DMSO and measured at 560 nm.

### Cell surface hydrophobicity (CSH) assay

Bacterial surface hydrophobicity was measured using the bacterial adhesion to hydrocarbon (BATH) assay as described in Hui & Dykes (2012). *P. aeruginosa* culture was collected at stationary phase and pelleted by centrifugation (NF800R, NUVE, Belgium) at 7,000 rpm for 10 min. This pellet was washed twice in phosphate buffer saline (PBS; pH 7.4), and then resuspended in PBS with PGPE (25 and 50 μg/ml) to OD _764_= 1.00. Same procedure was repeated with *P. aeruginosa* culture without PGPE, as a control. Each bacterial suspension was then incubated for 1 h at room temperature. A 2-ml sample of each suspension was collected and absorbance (A) at 764 nm was measured, using PBS as the blank. Next, 1 ml xylene (HiMedia, Mumbai) was added to the 2-ml cell suspension, and this mixture was vortexed for 2 min. The phases were then allowed to separate for 1 h. The absorbance of the aqueous phase (A _0_) was again determined. Results were expressed as % attachment to xylene = (1-A/A _0_) × 100.

### Determination of the effect of PGPE on antibiotic susceptibility of the test organism

**Figure 1.**
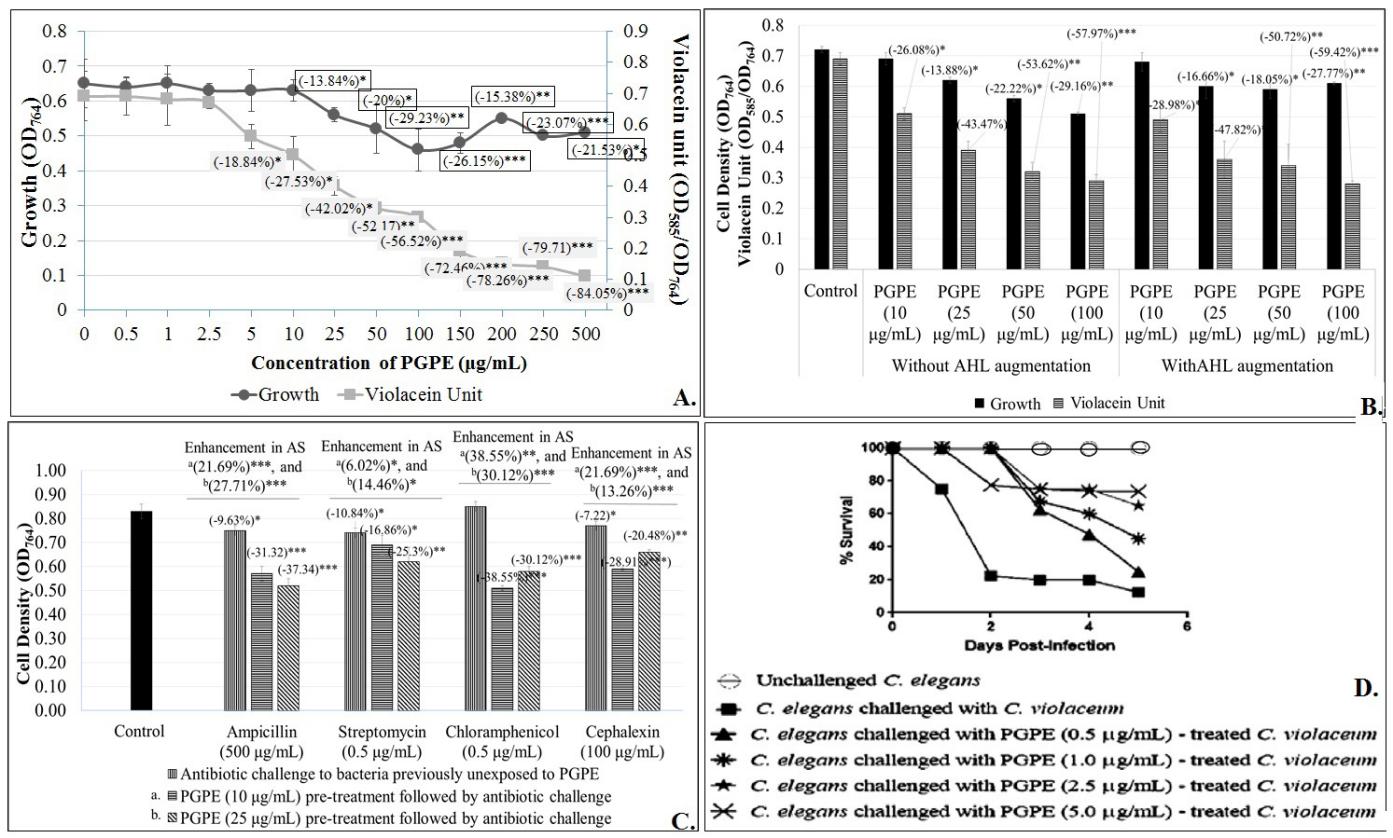
Effect of PGPE on *C. violaceum*. ‘Control’ in this figure is the vehicle control (0.5%v/v DMSO), which did not exert any effect on growth and violacein production of *C. violaceum*. ( **A**) Effect of PGPE on growth and QS- regulated violacein production in *C. violaceum*: Bacterial growth was measured as OD _764_; OD of violacein was measured at 585 nm, and Violacein Unit was calculated as the ratio OD _585_/OD _764_ (an indication of violacein production per unit of growth); Catechin (50 µg/ml) did not exert any effect on growth of *C. violaceum*, but inhibited violacein production by 47.69±0.03%. ( **B**) PGPE acts as a *signal-response inhibitor* against *C. violaceum.* ( **C**) PGPE-pre-treatment enhances susceptibility of *C. violaceum* to different antibiotics. ( **D**) PGPE-treatment attenuates virulence of *C. violaceum* towards *C. elegans*: Catechin (50 μg/ml) and ampicillin (500 μg/ml) employed as positive controls conferred 100% protection on worm population, and pre-treatment of bacteria with PGPE at concentrations (10, 25, 50 and 100 μg/ml) other than those shown in figure allowed 75%, 77.5%, 80%, and 75% worm survival, respectively; DMSO present in the ‘vehicle control’ at 0.5%v/v did not affect virulence of the bacterium towards *C. elegans;* DMSO (0.5%v/v) and PGPE at tested concentrations showed no toxicity towards the worm. * *p*<0.05, ** *p*<0.01, *** *p*<0.001; AS, antibiotic susceptibility; QS, quorum sensing; PGPE, *Punica granatum* peel extract.

**Figure 2.**
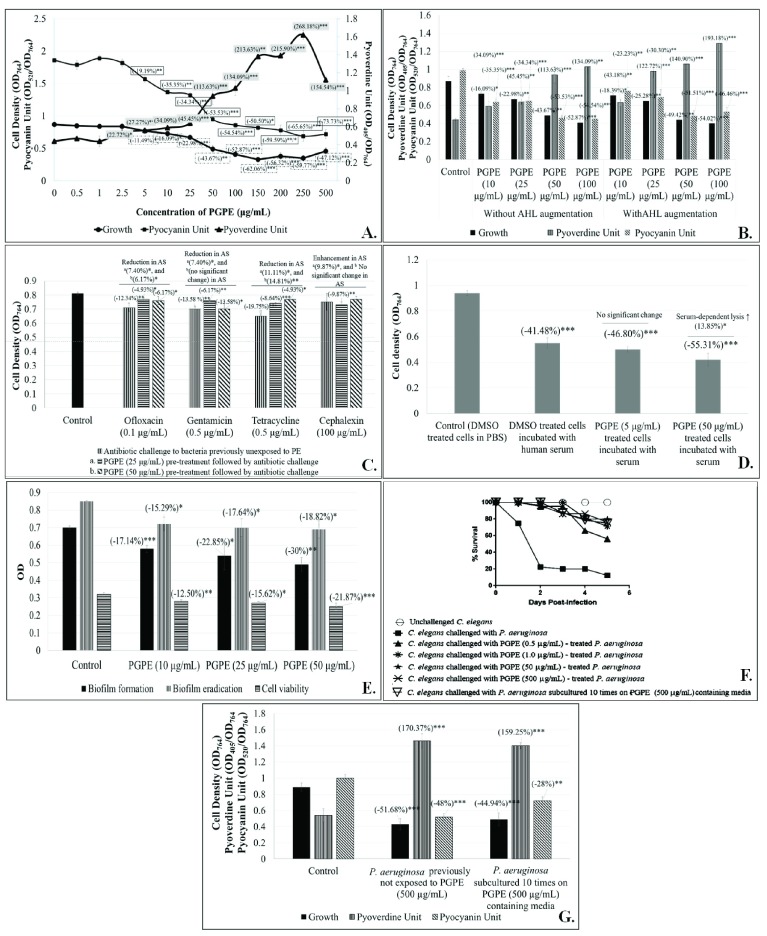
Effect of PGPE on various traits of *P. aeruginosa, in vitro*. ‘Control’ in this figure is the vehicle control (0.5%v/v DMSO), which did not exert any effect on growth and pigment production of *P. aeruginosa*. ( **A**) Effect of PGPE on growth and QS-regulated pigment production in *P. aeruginosa:* Bacterial growth was measured as OD _764_; OD of pyoverdine was measured at 405 nm, and Pyoverdine Unit was calculated as the ratio OD _405_/OD _764_ (an indication of pyoverdine production per unit of growth), Pyocyanin Unit was calculated as the ratio OD _520_/OD _764_ (an indication of pyocyanin production per unit of growth); Catechin (50 µg/ml) inhibited pyoverdine and pyocyanin production by 17.13±0.06% and 23.65±0.04% respectively, without affecting the bacterial growth. ( **B**) PGPE acts as a *signal-response inhibitor* against *P. aeruginosa.* ( **C**) *P. aeruginosa* challenged with antibiotics following pre-treatment with PGPE. ( **D**) PGPE modulates susceptibility of *P. aeruginosa* to lysis by human serum: ‘Control’ in serum-dependent lysis assay was PGPE-unexposed cells of *P. aeruginosa* incubated with human serum. ( **E**) Effect of PGPE on *P. aeruginosa* biofilm formation, eradication, and viability. Crystal violet assay was performed to measure biofilm formation, and biofilm eradication, followed by the measurement of OD at 580 nm; Cell viability in biofilm was estimated through MTT assay, wherein OD was measured at 560 nm. ( **F**) PGPE-treatment reduces the virulence of *P. aeruginosa* towards *C. elegans*: Catechin (50 μg/ml) and gentamicin (0.1 μg/ml) employed as a positive controls conferred 100% and 80% protection on worm population respectively. Pre-treatment of bacteria with PGPE at concentrations (2.5, 5, 10, 25, and 100 μg/ml) other than shown in figure allowed 73.75, 75, 77.5, 77.5, and 75% worm survival respectively; DMSO present in the ‘vehicle control’ at 0.5%v/v did not affect virulence of the bacterium towards *C. elegans;* DMSO (0.5%v/v) and PGPE at tested concentrations showed no toxicity towards the worm. ( **G**) Effect of PGPE on *P. aeruginosa* growth, Pyoverdine Unit, and Pyocyanin Unit remained unaltered after repeated exposure to PGPE (500 μg/ml). *p<0.05 , **p<0.01 , ***p<0.001. AS, antibiotic susceptibility; QS, quorum sensing; PGPE, *Punica granatum* peel extract.

After *in vitro* assessment of QS modulatory (QSM) property of the test formulation, effect of this PGPE on antibiotic susceptibility of the test pathogen was investigated. The bacterial cells pre-treated with PGPE were subsequently challenged with sub-MIC concentration of antibiotic. All the antibiotics were procured from HiMedia, Mumbai. Names and concentrations of the antibiotics used can be seen in Figure 1c, Figure 2c, Figure 5c and Figure 7e.

### *In vivo* assay

*In vivo* efficacy of the PGPE was evaluated using the nematode worm *Caenorhabditis elegans* as the model host, using the method described by Eng & Nathan (2015) with some modification. This worm was maintained on Nematode Growing Medium (NGM; 3 g/l NaCl, 2.5 g/l peptone, 1 M CaCl _2_, 1 M MgSO _4_, 5 mg/ml cholesterol, 1 M phosphate buffer of pH 6, 17 g/l agar-agar) with *Escherichia coli* OP50 (procured from LabTIE B.V., JR Rosmalen, the Netherlands) as the feed. Worm population to be used for the *in vivo* assay was kept on NGM plates not seeded with *E. coli* OP50 for three days, before being challenged with the test pathogen.

Test bacterium was incubated with the PGPE for 22–24h. Following incubation, OD _764_ of the culture suspension was equalized to that of the DMSO control. 100 µl of this bacterial suspension was mixed with 900 µl of the M9 buffer containing 10 worms (L3–L4 stage). This experiment was performed in 24-well (sterile, non-treated) polystyrene plates (HiMedia), and incubation was carried out at 22°C. Number of live vs. dead worms was counted everyday till five days by putting the plate (with lid) under light microscope (4X). Standard antibiotic- (ampicillin 500 µg/ml; gentamicin 0.1 µg/ml) and catechin- (50 µg/ml) treated bacterial suspension were used as positive control. Straight worms were considered to be dead. On last day of the experiment, when plates could be opened, their death was also confirmed by touching them with a straight wire, wherein no movement was taken as confirmation of death.

### Investigating whether the bacterium becomes resistant to PGPE upon repeated exposure

*P. aeruginosa* was subcultured on PGPE (500 μg/ml)-containing agar medium 10 times, and this PGPE-habituated culture was then challenged with PGPE (500 μg/ml) in liquid media for broth dilution assay, wherein bacterial cell density and pigment production were quantified as detailed in preceding sections. Virulence of this PGPE-habituated culture towards *C. elegans* was also assessed using the same methodology as described above under the heading ‘ *In vivo* assay’.

### Chromatographic analysis

Chromatographic fingerprint of PGPE was generated through HPLC (Shimandzu Lab Solutions) by running the extract through a C18 column (Shimandzu) for 75 min. Mobile phase consisted of a mixture of trichloroacetic acid (0.05%, w/v) in water, and trichloroacetic acid (0.05%, w/v) in methanol, wherein proportion of latter was varied over a gradient of 10–80%. Flow rate was 1 ml/min. Detection wavelength of the UV detector was set at 258 nm. Punicalagin A and punicalagin B (both at 100 ppm; Sigma Aldrich) were used as standard compounds.

### Molecular docking

Structures of the pure compounds were downloaded from PubChem: catechin (PubChem CID: 73160), gallic acid (GA) (PubChem CID: 370), quercetin (PubChem CID: 5280343), ellagic acid (PubChem CID: 5281855), cinnamic acid (PubChem CID: 444539), and chlorogenic acid (PubChem CID: 1794427). Structure of the bacterial target proteins was downloaded from the Protein Data Bank (PDB): CviR of *C. violaceum* (PDB ID 3QP4), LasR (PDB ID 2UV0), and PqsR (PDB ID 4JVC) of *P. aeruginosa*. AutoDock Vina ( Trott & Olson, 2010) was used for docking studies, Discovery Studio Visualizer 4.0.1 was used to study protein-ligand interactions, Pymol viewer (version v1.3r1) was used to convert protein-ligand docked structures from .pdbqt to .pdb format, and MglTools was used to prepare protein and ligand files for docking. 

### Statistical analysis

All the *in vitro* experiments were performed in triplicate, and measurements are reported as mean ± standard deviation (SD) of three independent experiments. *In vivo* experiments were run in four replicates. Statistical significance of the data was evaluated by applying *t*-test using Microsoft Excel. *p* values ≤0.05 were considered to be statistically significant.

## Results

### *C. violaceum* 

*C. violaceum* was challenged with the PGPE at 0.5–500 μg/ml ( Figure 1A). PGPE had a negative effect on bacterial growth only at concentrations ≥25 μg/ml; however, an increase in concentration could not inhibit growth to any greater extent. In fact, statistically all the concentrations in the range 50–500 μg/ml had equivalent effect on *C. violaceum* growth. On the other hand, production of the QS-regulated pigment violacein was sensitive to PGPE at lesser concentrations (i.e. at ≥5 μg/ml), and the inhibitory effect increased with increase in concentration of the extract. PGPE can be said to exert a purely QSM effect at 5–10 μg/ml, as at these concentrations, it could inhibit pigment production significantly without inhibiting *C. violaceum* growth.

Once the QS-inhibitory potential of PGPE against *C. violaceum* was confirmed, we experimented to know whether it acts as a signal-supply inhibitor or signal-response inhibitor. To know this, we added AHL (2% v/v; 6 h post-inoculation) into quorum inhibited culture of *C. violaceum* growing in the presence of PGPE (10–100 μg/ml). Following AHL (signal) augmentation, the quorum inhibitory effect of PGPE on violacein production was not reversed ( Figure 1B), indicating that PGPE acts as a signal-response inhibitor.

When PGPE-treated *C. violaceum* culture was subsequently challenged with sub-MIC concentrations of four antibiotics belonging to four different classes, extract pre-treatment was found to enhance susceptibility of this bacterium to all these antibiotics. However, pre-treatment with higher (25 μg/ml) PGPE concentration was found to be no better than the lesser (10 μg/ml) concentration, with respect to making bacteria more susceptible to given antibiotic, except in the case of ampicillin ( Figure 1C).

PGPE was found to curb haemolytic potential of *C. violaceum* in a dose-dependent fashion, wherein this extract at 100 μg/ml could reduce haemolysis by ~36% ( Table 1). This extract could also reduce the catalase activity of *C. violaceum* marginally; however, the effect on this oxidative stress response enzyme activity did not increase with increase in PGPE concentration ( Table 1).

**Table 1.** Effect of PGPE on catalase and haemolytic activity of test bacteria.

Organism	Concentration of extract, μg/ml	% Change in catalase activity (mean ± SD)	% Inhibition in Hemolysis (Mean ± SD)
*C. violaceum*	10	-4.65 * ± 2.46	8.83 * ± 3.46
	25	-5.81 * ± 3.28	22.09 ** ± 2.40
	50	-8.13 * ± 1.64	28.72 ** ± 3.79
	100	-11.62 * ± 6.57	35.91 *** ± 3.02
*P. aeruginosa*	10	0.79 ± 0.62	-9.19 ± 6.27
	25	0.78 ± 0.44	15.47 * ± 3.33
	50	-1.77 ± 0.47	22.61 * ± 4.89
	100	-2.56 * ± 0.15	33.33 ** ± 3.83

* *p*<0.05, ** *p*<0.01, *** *p*<0.001; Catalase assay was done by monitoring disappearance of H _2_O _2_ at 240 nm; Hemoglobin concentration was measured at OD _540; _ DMSO in ‘vehicle control’ tube had no effect on catalase and haemolytic activity of any of the three bacteria; Chloramphenicol (0.5 μg/ml) enhanced catalase activity of *C. violaceum* by 11.23%*.± 0.01; Tetracycline (0.5 μg/ml) inhibited catalase activity of *P. aeruginosa* by 21.51%* ± 0.02.

*In vitro* demonstration of the QSM potential of PGPE was followed by assessment of its anti-infective potential *in vivo*, employing *C. elegans* as the model host, wherein PGPE was found to confer survival benefit on *C. elegans* upon challenge with *C. violaceum* at all the concentrations (0.5–100 μg/ml) tested ( Figure 1D). Notably, even the concentrations (0.5–2.5 μg/ml) having no significant *in vitro* effect on *C. violaceum* growth and pigment production, were found to be effective *in vivo*. Maximum *in vivo* benefit was obtained at 5 μg/ml PGPE concentration, and worm survival percentage at any of the higher concentrations were statistically no better than that obtained at 5 μg/ml. Onset of death in the worm population was also delayed by 2 days (i.e. death started on first day-post infection in control wells, as against on third day in experimental wells) when the challenger bacteria were pre-treated with PGPE. Movement of the surviving worm in ‘vehicle control’ wells was slowed down, whereas worms surviving in the ‘experimental’ wells showed normal movement. Further, worms in the experimental well corresponding to 100 μg/ml PGPE could also generate progeny worms, which were not observed in any other well.

### *P. aeruginosa* 

*P. aeruginosa* was challenged with PGPE at 0.5–500 μg/ml, wherein growth of this bacterium got notably inhibited 50 μg/ml onwards. Pyocyanin production was affected negatively from 5 μg/ml onwards in a concentration-dependent manner, whereas pyoverdine production was enhanced from 2.5 μg/ml onwards in a largely concentration-dependent fashion except at 500 μg/ml ( Figure 2A). When AHL was added exogenously to the quorum-modulated *P. aeruginosa* culture, the inhibitory effect of PGPE on pyocyanin and its stimulatory effect on pyoverdine production was not reversed ( Figure 2B) indicating that PGPE acts as a signal-response inhibitor against this pathogen.

Effect of PGPE pre-treatment on antibiotic susceptibility of *P. aeruginosa* was also investigated. However, no major changes were observed in bacterial susceptibility to four different test antibiotics, when challenged with these antibiotics following PGPE exposure ( Figure 2C). This extract was not found to alter catalase activity much; however, haemolytic potential of *P. aeruginosa* was notably affected 25 μg/ml onwards ( Table 1). PGPE at 50 μg/ml could enhance (by 13.55%; *p=*0.03) susceptibility of this bacterial pathogen to lysis by human serum ( Figure 2D).

PGPE was able to reduce *P. aeruginosa* biofilm formation up to 17–30%, when tested at 10–50 μg/ml ( Figure 2E). When this extract was applied on pre-formed biofilm, it could eradicate the biofilm by ~15–19%; and biofilm viability (metabolic activity as measured by MTT assay) was reduced by ~13–22%. However, whether this observed reduction in viability is due to lesser number of cells, or some other reason, remains to be investigated. Reduced biofilm formation by *P. aeruginosa* in presence of PGPE may partly be due to this extract’s ability to reduce CSH marginally, which is an important determinant of biofilm forming ability in bacteria ( Hui & Dykes, 2012). Xylene adherence of *P. aeruginosa* treated with PGPE at 25 and 50 μg/ml was found to be reduced by 14.19% (p<0.05; % adherence=44.74) and 7.82% (p<0.05; % adherence =38.37) respectively, against 30.55% adherence of control.

*In vivo* assay did confirm the anti-infective potential of PGPE against *P. aeruginosa*. At concentrations as low as 0.5–1 μg/ml, it could support overall survival of the worm population up to 56–71% in face of *P. aeruginosa* challenge, as against only 12.5% worm survival in control population. Higher PGPE concentrations did not offer any better protection to *C. elegans* against *P. aeruginosa* ( Figure 2F). Notably, *in vitro* experiments showed PGPE to have no effect on *P. aeruginosa* growth below 5 μg/ml, and no effect on its QS-regulated pigments below 2.5 μg/ml; whereas PGPE at 0.5–1 μg/ml was able to confer significant survival benefit on *C. elegans* against *P. aeruginosa* challenge. Onset of death in worm population was also delayed by 1–3 days, when *P. aeruginosa* was exposed to PGPE before being allowed to infect *C. elegans*.

After confirming the anti-infective potential of PGPE against *P. aeruginosa*, we investigated whether this bacterium can develop resistance to PGPE upon repeated exposure to this extract. We sub-cultured *P. aeruginosa* on PGPE (500 μg/ml) containing agar medium 10 times, and this PGPE-habituated culture was then challenged with PGPE (500 μg/ml) in liquid media for broth dilution assay, wherein effect of PGPE on growth and pigment production in *P. aeruginosa* was not found to be much different than that on *P. aeruginosa* with no previous PGPE exposure ( Figure 2G). Similarly, there was no difference in the virulence of *P. aeruginosa* towards *C. elegans*, irrespective of whether it was previously habituated to PGPE ( Figure 2F). 

**Figure 3.**
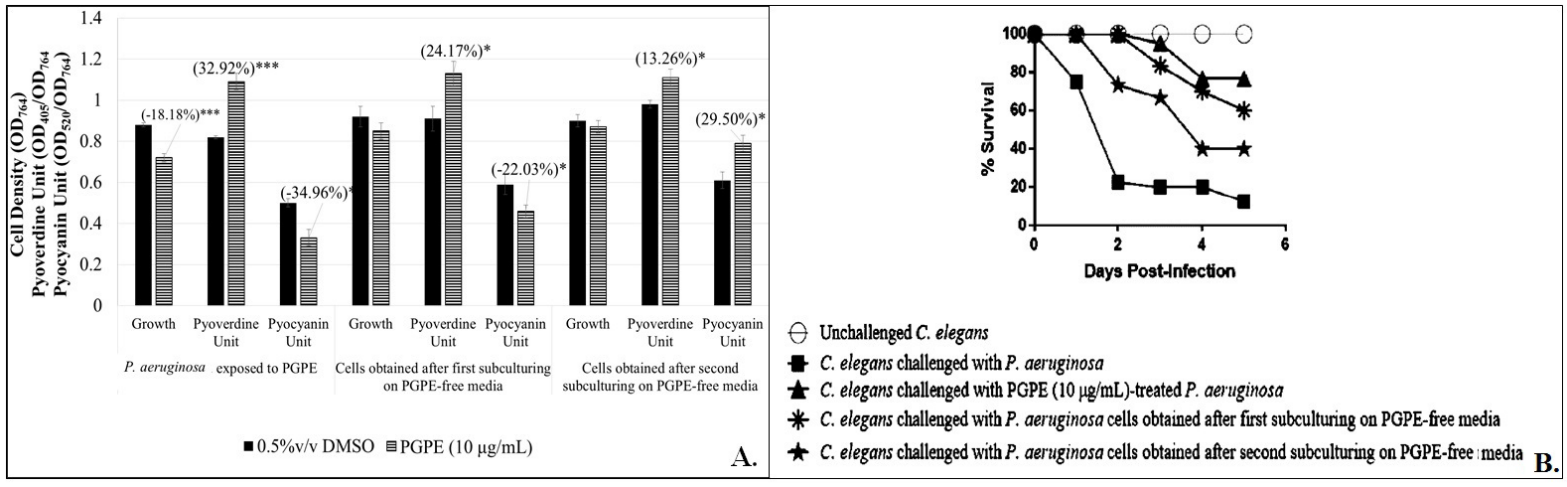
Post extract effect of PGPE (10 μg/ml) on *P. aeruginosa*. ‘Control’ in this figure is the vehicle control (0.5%v/v DMSO), which did not exert any effect on growth and pigment production of *P. aeruginosa.* ( **A**) Effect of PGPE on growth and QS-regulated pigment production in *P. aeruginosa* after subculturing of cells in PGPE-free media. Bacterial growth was measured as OD _764_; OD of pyoverdine was measured at 405 nm, and Pyoverdine Unit was calculated as the ratio OD _405_/OD _764_ (an indication of pyoverdine production per unit of growth), Pyocyanin Unit was calculated as the ratio OD _520_/OD _764_ (an indication of pyocyanin production per unit of growth). ( **B**) PGPE-treatment reduces the virulence of *P. aeruginosa* towards *C. elegans* even after subculturing of cells in PGPE-free media. Catechin (50 μg/ml) and gentamicin (0.1 μg/ml) employed as a positive controls conferred 100% and 80% protection on worm population respectively; DMSO present in the ‘vehicle control’ at 0.5%v/v did not affect virulence of the bacterium towards *C. elegans;* DMSO (0.5%v/v) and PGPE at tested concentrations showed no toxicity towards the worm. *p<0.05 , **p<0.01 , ***p<0.001; AS, antibiotic susceptibility; QS, quorum sensing; PGPE, *Punica granatum* peel extract.

With *P. aeruginosa,* we did an additional experiment to investigate whether the daughter cells of PGPE-exposed *P. aeruginosa* bear any attenuation of their virulence owing to their parent cells being exposed to PGPE. When the PGPE-exposed *P. aeruginosa* was subsequently subcultured on PGPE-free media, pigment production in cells obtained after first such subculturing was still altered in a pattern similar to the PGPE-exposed parent culture ( Figure 3A). Though QS-regulated pigment production remained deviated from ‘control’ upon second subculturing too, it did not follow the same pattern, as observed with cells obtained after first subculturing on PGPE-free media. With each subculturing, virulence of *P. aeruginosa* towards *C. elegans* approached nearer to that of ‘control’, but it still did not got equivalent to ‘control’ ( Figure 3B), clearly suggesting that effect of PGPE-treatment was not completely absent even from the progeny cells (who never were directly exposed to PGPE), whose parent cells received single PGPE-exposure. This phenomenon of long-lasting effect after single-time exposure to the antimicrobial agent is described as ‘post-antimicrobial effect’. Here, in case of a plant extract, we prefer to call it post-extract effect (PEE), which refers to the persistent suppression of one or more bacterial traits (e.g. growth/virulence, etc.) after one-time-exposure to antimicrobial agents, and may last for many hours depending on the concentration of test agent and the susceptibility of the target bacterium ( Pfaller *et al.,* 2004; Ramadan *et al.,* 1995; Ramanuj *et al.,* 2012). During actual animal/human infections, such phenomenon can be of high significance, as though the infectious bacteria may multiply inside the host system, their progenies may not have the virulence at par with the parent cells.

### Characterization of PGPE

**Figure 4.**
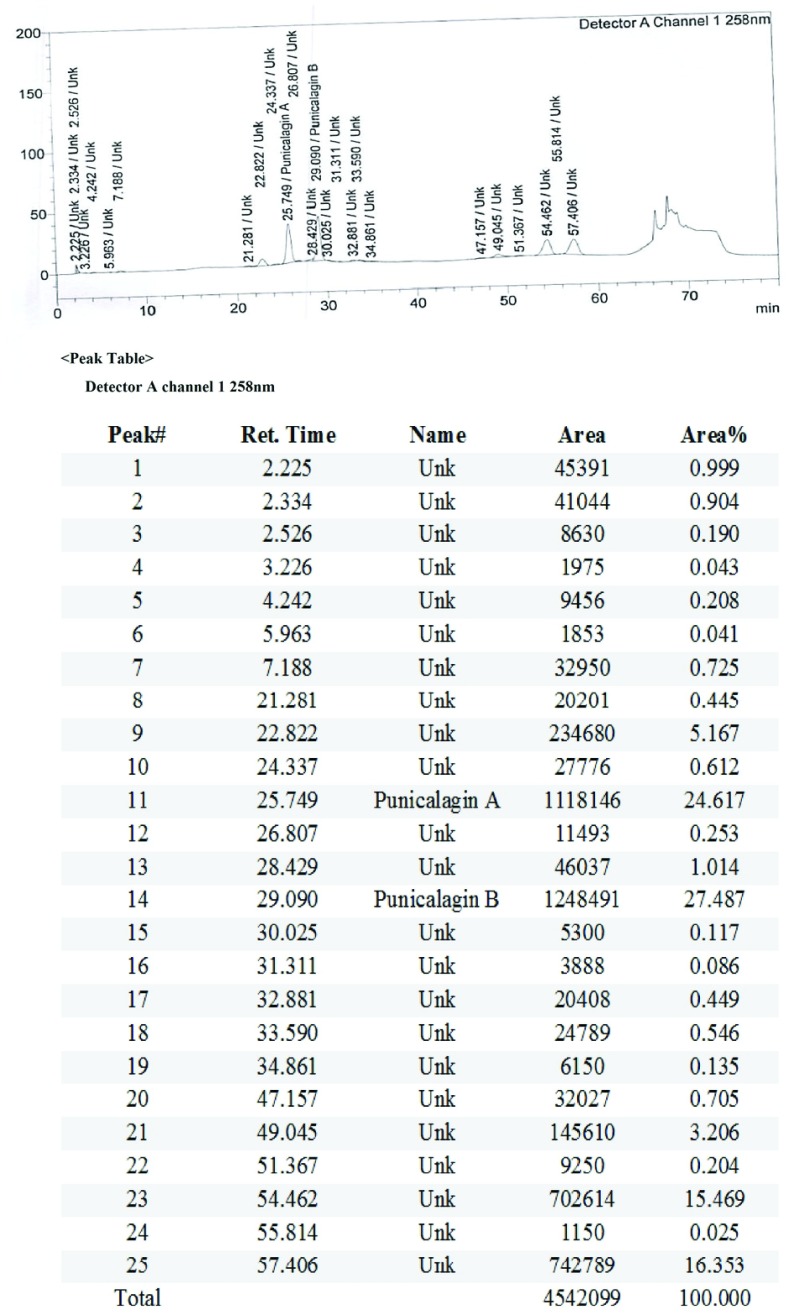
HPLC profile of PGPE. Peak 11 and 14 occupied 24.61% and 27.48% of total area, which corresponds to Punicalagin A and B, respectively. PGPE, *Punica granatum* peel extract.

Once *in vitro* and *in vivo* anti-pathogenic potential of PGPE was confirmed, we proceeded to chromatographic fingerprinting of this extract. The resultant chromatogram is shown in Figure 4. Since punicalagin is one of the most widely reported bioactive metabolite from *P. granatum*, we quantified its amount in our extract, which was found to be present at 6.6%. Earlier we had analyzed another pomegranate fruit extract marketed as ‘Pomella’ by Pharmanza Herbal Pvt. Ltd., which contained not less than 30% punicalagin for its QS-modulatory potential (extended data, Figure S1A) ( Joshi *et al.,* 2018). Though there were no major differences with respect to the *in vitro* effect on growth and pyocyanin production by *P. aeruginosa*, pyoverdine production was affected much more by Pomella than PGPE. However, *in vivo* efficacy of PGPE was bit better than that of Pomella; PGPE at 0.5–50 μg/ml allowed 56–80% worm survival, as against 55–65% worm survival supported by ‘Pomella’ at identical concentrations (extended data, Figure S1B) ( Joshi *et al.,* 2018). From this, we may speculate punicalagin not to be the major constituent responsible for *P. granatum* extract’s anti-pathogenic activity. Against *C. violaceum* too, except at 250 μg/ml, effect of PGPE on violacein production was statistically not different from that of Pomella, with latter exerting a bit more inhibitory effect on growth of this bacterium (extended data, Figure S1C) ( Joshi *et al.,* 2018). However, role of punicalagins cannot be completely ruled out, as they are known to be metabolized inside human system to urolithins, and latter has been reported to be capable of exerting anti-QS effect ( Giménez-Bastida *et al.,* 2012). Larrosa *et al.* (2010) suggested the possibility of urolithin-A being the most active anti-inflammatory compound derived from pomegranate ingestion in healthy subjects.

**Figure 5.**
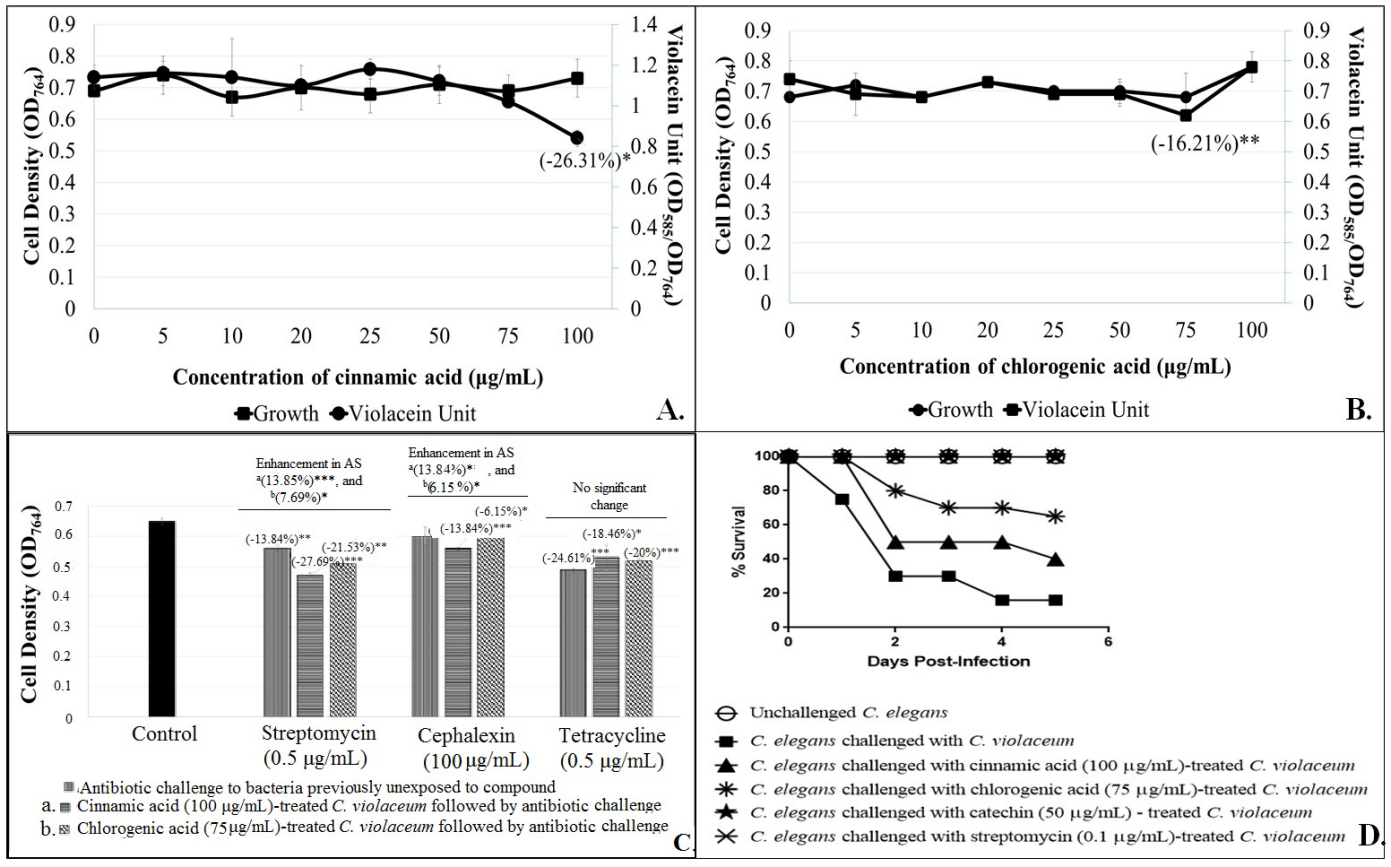
Effect of cinnamic acid and chlorogenic acid on *C. violaceum*. ‘Control’ in this figure is the vehicle control (0.5%v/v DMSO), which did not exert any effect on growth and violacein production of *C. violaceum.* ( **A**) Effect of cinnamic acid on growth and violacein production in *C. violaceum*. Bacterial growth was measured as OD _764_; OD of violacein was measured at 585 nm, and Violacein Unit was calculated as the ratio OD _585_/OD _764_ (an indication of violacein production per unit of growth). ( **B**) Effect of chlorogenic acid on growth and violacein production in *C. violaceum*. Bacterial growth was measured as OD _764_; OD of violacein was measured at 585 nm, and violacein unit was calculated as the ratio OD _585_/OD _764_ (an indication of violacein production per unit of growth). ( **C**) Pre-treatment of *C. violaceum* with chlorogenic acid and cinnamic acid enhances its susceptibility to streptomycin and cephalexin. ( **D**) Cinnamic acid, chlorogenic acid, and catechin reduced virulence of *C. violaceum* towards *C. elegans*. Catechin (50 μg/ml) and ampicillin (500 μg/ml) employed as positive controls conferred 100% protection on worm population. DMSO present in the ‘vehicle control’ at 0.5%v/v did not affect virulence of the bacterium towards *C. elegans;* DMSO (0.5%v/v) and compounds at tested concentrations showed no toxicity towards the worm. * *p*<0.05, ** *p*<0.01, *** *p*<0.001. AS, antibiotic susceptibility; QS, quorum sensing.

### QSM potential of *P. granatum* peel phytocompounds in pure form

Other than punicalagin, pomegranate peel extracts have been reported to contain pyrogallol, catechin, rutin, cinnamic acid, benzoic acid, chlorogenic acid, acacetin, ferulic acid, kampferol, genistein ( Ali *et al.,* 2014), coumaric acid ( Khan *et al.,* 2017) tannins (punicalin, pedunculagin, GA and ellagic acid) ( Ismail *et al.,* 2012; Pagliarulo *et al.,* 2016), and quercetin ( Saxena *et al.,* 2017). To gain more insight, we docked all these reported compounds against LuxR analogues of *C. violaceum* and *P. aeruginosa*, against which our extract was found to act as signal-response inhibitor. Results of this molecular docking exercise are presented in Table 3. The amino acid residues involved in this on-screen ligand-receptor binding, for compounds used in wet-lab study, are shown in extended data, Table S1 ( Joshi *et al.,* 2018).

***Pure compounds against C. violaceum.*** Against the LuxR analogue of *C. violaceum*, docking scores higher than that of its natural ligand (C6-HSL) were obtained for acacetin, catechin, and quercetin. However, for further *in vitro* experiments, we did not set any cut-off value with respect to docking score, instead we experimented with all those pure compounds from those listed in Table 2, which were available in our lab viz. catechin, quercetin, chlorogenic acid, cinnamic acid, and GA. Cinnamic acid had no effect on *C. violaceum* growth and pigment production in the concentration range 5–100 μg/ml, except 26% reduction in violacein production at 100 μg/ml ( Figure 5A). Hence all subsequent experiments with cinnamic acid were performed at 100 μg/ml. Cinnamic acid was able to enhance *C. violaceum* susceptibility to streptomycin by 11.94%, but had no effect on its susceptibility to cephalexin and tetracycline ( Figure 5C). Haemolytic potential and catalase activity of *C. violaceum* were curbed to a smaller (8.47% and 2.68%, respectively), albeit statistically significant extent ( Table 3). This phenylpropanoid metabolite was also able to confer some survival benefit (24% lesser worm death) on *C. elegans* ( Figure 5D). Cinnamic acid induced violacein inhibition was not reversed upon exogenous supply of AHL (extended data, Figure S2) ( Joshi *et al.,* 2018), indicating its mode of QS-inhibition to be signal-response inhibition. 

**Table 2.** Docking score of compounds with various targets.

Compound	PubChem CID	Binding affinity (kcal/mol)		
		*C. violaceum* (CviR)	*P. aeruginosa* (LasR)	*P. aeruginosa* (PqsR)
(Natural ligand) Autoinducer ^#^	C6-HSL: 3462373 C12-HSL: 3246941 PQS: 2763159	-7.6	-7.2	-6.2
Acacetin	5280442	-9.0	-5.9	-6.9
Benzoic acid	243	-7.1	-6.1	-5.0
Catechin	73160	-8.5	-9.8	-6.8
Chlorogenic acid	1794427	-7.3	-5.7	-7.5
Cinnamic acid	444539	-7.1	-6.9	-4.7
Coumaric acid	637542	-6.4	-7.1	-5.3
Ellagic acid	5281855	-6.8	-8.8	-6.6
Ferulic acid	445858	-6.4	-7.5	-5.7
Gallic acid	370	-5.9	-6.3	-5.8
Genistein	5280961	-6.3	-9.4	-6.5
Kaempferol	5280863	-6.8	-7.2	-6.9
Pedunculagin	442688	-7.6	-6.4	-8.1
Punicalagin	44584733	-7.0	-7.4	-9.3
Punicalin	5388496	-7.4	-6.9	-8.3
Pyrogallol	1057	-5.2	-6.1	-5.1
Quercetin	5280343	-8.5	-9.9	-7.2
Rutin	5280805	-7.3	-6.7	-8.2

^#^C6-HSL autoinducer of *C. violaceum*, C12-HSL autoinducer of Las QS system of *P. aeruginosa*, and PQS quinolone signal of PQS system of *P*. *aeruginosa* were used for docking as control.

**Table 3.** Effect of pure compounds on catalase and haemolytic activity of test bacteria.

Organism	Compound	Concentration of extract, µg/ml	Change in catalase activity, % †	Inhibition in haemolysis, % †
*C. violaceum*	Gallic acid	100	11.53 *** ± 1.51	14.28 * **±**4.73
		150	12.30 *** ± 0.65	22.85 *** ± 5.22
		200	10.25 *** ± 0.51	18.57 * ± 2.78
	Quercetin	100	1.79 * ± 0.30	9.52 ** ± 3.55
		150	2.09 * ± 0.75	13.09 ** ± 1.11
		200	2.39 * ± 0.62	19.04 *** ± 2.61
	Cinnamic acid	100	-2.68 * ± 1.47	8.47 * ± 4.90
	Chlorogenic acid	75	-3.84 * ± 0.64	10.16 * ± 5.80
	Catechin	50	4.93 *** ± 1.00	17.54 * ± 3.91
*P. aeruginosa*	Gallic acid	100	0.31 ± 0.67	9.64 ± 5.59
		150	0.62 ± 0.47	9.41 ± 1.18
		200	0.62 ± 0.45	9.52 ± 2.10
	Quercetin	100	-2.84 ** ± 0.50	2.82 * ± 0.009
		150	-1.26 * ± 0.30	9.73 *** ± 0.30
		200	-2.61 *** ± 0.47	12.34 ** ± 1.97
	Cinnamic acid	100	1.18 ± 0.41	19.11 * ± 6.23
	Chlorogenic acid	75	1.47 * ± 0.83	35.29 *** ± 3.70
	Catechin	50	-9.00 ** ± 1.91	15.06 *** ± 3.66

* *p*<0.05, ** *p*<0.01, *** *p*<0.001. †Mean ± SD. Catalase assay was done by monitoring disappearance of H _2_O _2_ at 240 nm; Hemoglobin concentration was measured at OD _540_; DMSO in ‘vehicle control’ tube had no effect on catalase and haemolytic activity of any of the three bacteria; Chloramphenicol (0.5 μg/mL) enhanced catalase activity of *C. violaceum* by 11.23%*. ± 0.01; Tetracycline (0.5 μg/mL) inhibited catalase activity of *P. aeruginosa* by 21.51%* ± 0.02.

Chlorogenic acid could not affect *C. violaceum* growth and pigment production at any of the test concentrations (5–100 μg/ml), except 16.21% inhibition of violacein production at 75 μg/ml ( Figure 5B). Notably, violacein production was not affected at higher concentration (i.e. 100 μg/ml). Hence, all subsequent experiments with chlorogenic acid were performed at 75 μg/ml. At this concentration chlorogenic acid could make *C. violaceum* 8.91% more susceptible to streptomycin; however, it did not alter this bacterium’s susceptibility to cephalexin and tetracycline ( Figure 5C). Chlorogenic acid was also able to inhibit catalase activity and haemolytic activity of *C. violaceum* by 3.84% and 10.16% respectively ( Table 3). It could also offer a sizable (49%) survival benefit to *C. elegans*, when challenged with *C. violaceum* ( Figure 5D). AHL augmentation of the *C. violaceum* culture experiencing chlorogenic acid induced QS inhibition, did not result in reversal of this inhibition (extended data, Figure S2) ( Joshi *et al.,* 2018), indicating this plant metabolite to act as a signal-response inhibitor.

**Figure 6.**
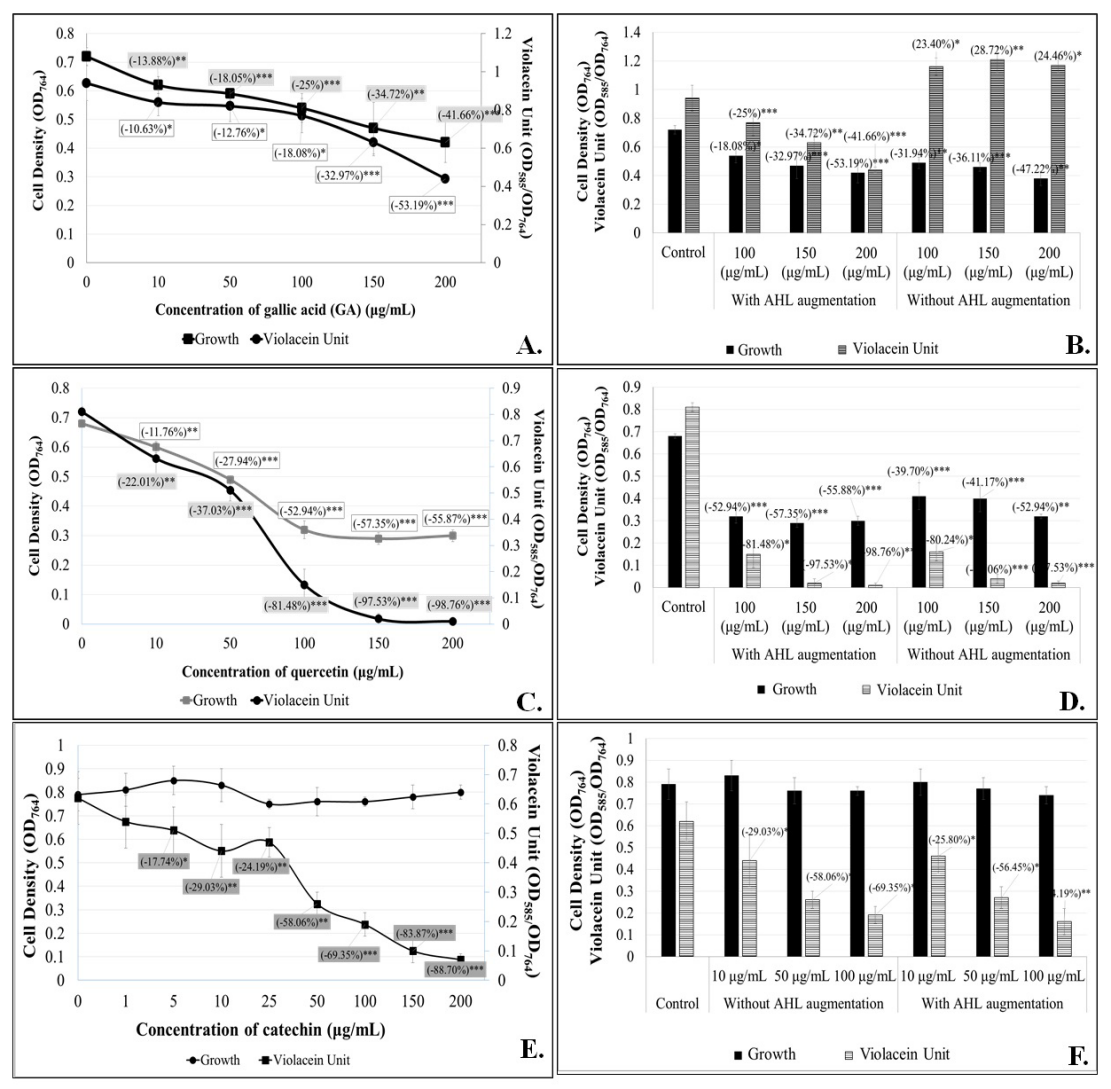
Effect of GA, quercetin, and catechin on *C. violaceum*. Bacterial growth was measured as OD _764_; OD of violacein was measured at 585 nm, and Violacein Unit was calculated as the ratio OD _585_/OD _764 _(an indication of violacein production per unit of growth) ‘Control’ in this figure is the vehicle control (0.5%v/v DMSO), which did not exert any effect on growth and violacein production of *C. violaceum*. ( **A**) Effect of gallic acid on growth and violacein production in *C. violaceum.* ( **B**) GA acts as a signal-supply inhibitor. ( **C**) Effect of quercetin on growth and violacein production in *C. violaceum.* ( **D**) Quercetin acts as a signal-response inhibitor. ( **E**) Effect of catechin on growth and violacein production in *C. violaceum.* ( **F**) Catechin acts as a signal-response inhibitor. *p<0.05, **p<0.01, ***p<0.001. QS, quorum sensing; GA, gallic acid.

GA was tested at 10–200 μg/ml for its potential quorum modulatory action against *C. violaceum*, and it was found to inhibit the growth of this bacterium in a dose-dependent fashion ( Figure 6A). Violacein production was negatively affected from 100 μg/ml onwards, and this inhibition was reversed upon external AHL supply ( Figure 6B). Since GA was indicated to act as a signal-supply inhibitor, we docked it against the LuxI analogue of *C. violaceum* i.e. CviI, the enzyme responsible for AHL synthesis, wherein the docking score was found to be -6.9 kcal/mol. At all test concentrations GA was able to induce catalase activity marginally.

**Figure 7.**
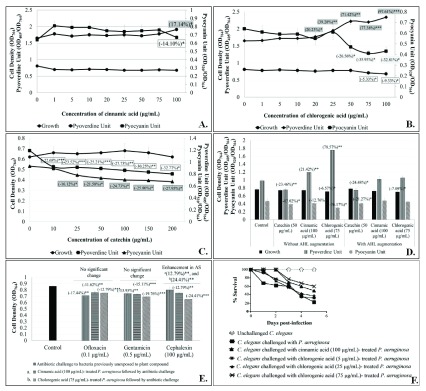
Effect of pure compounds on QS-regulated traits of *P. aeruginosa.* Bacterial growth was measured as OD _764_; OD of pyoverdine was measured at 405 nm, and Pyoverdine Unit was calculated as the ratio OD _405_/OD _764 _(an indication of pyoverdine production per unit of growth), Pyocyanin Unit was calculated as the ratio OD _520_/OD _764_ (an indication of pyocyanin production per unit of growth). ‘Control’ in this figure is the vehicle control (0.5%v/v DMSO), which did not exert any effect on growth and pigment production of *P. aeruginosa.* ( **A**) Effect of cinnamic acid on growth and QS-regulated pigment production in *P. aeruginosa.* ( **B**) Effect of chlorogenic acid on growth and QS-regulated pigment production in *P. aeruginosa.* ( **C**) Effect of catechin on growth and QS-regulated pigment production in *P. aeruginosa.* ( **D**) Cinnamic acid and chlorogenic acid act as *signal-supply inhibitors* against *P. aeruginosa*, whereas catechin acts as a *signal-response inhibitor.* ( **E**) *P. aeruginosa* receiving pre-incubation with cinnamic acid or chlorogenic acid becomes more susceptible to cephalexin. ( **F**) Pre-treatment of *P. aeruginosa* with cinnamic acid or chlorogenic acid reduces its virulence towards *C. elegans:* Catechin (50 μg/ml) and gentamicin (0.1 μg/ml) employed as positive controls conferred 100% and 80% protection on worm population respectively; DMSO present in the ‘vehicle control’ at 0.5%v/v did not affect virulence of the bacterium towards *C. elegans;* DMSO (0.5%v/v) and compounds at tested concentrations showed no toxicity towards the worm. * *p*<0.05, ** *p*<0.01, *** *p*<0.001; AS, Antibiotic susceptibility; QS, Quorum sensing.

 When *C. violaceum* was challenged with quercetin (10–200 μg/ml), its growth was negatively affected, and violacein production even more so. Concentration of 150 μg/ml quercetin was able to achieve almost complete inhibition of violacein production ( Figure 6C), and this inhibition was not reversible in response to exogenous AHL augmentation ( Figure 6D), indicating quercetin to act as a signal-response inhibitor. This observation also matches with the docking score (-8.5 Kcal/mol) of quercetin against CviR, which is second highest amongst all the docked compounds. Quercetin was also able to curb the catalase activity of *C. violaceum*, maximum inhibition being observed at 150 μg/ml.

 Catechin had no effect on *C. violaceum* growth till 200 μg/ml, whereas its quorum-inhibitory effect started from 10 μg/ml onwards. Hence, catechin can be said to have a purely quorum-inhibitory effect on *C. violaceum* ( Figure 6E), and its inhibitory effect on violacein production was not reversed upon AHL augmentation, indicating it to be acting as a signal-response inhibitor ( Figure 6F). This compound could notably reduce haemolytic potential of *C. violaceum*, and also had little but statistically significant effect on catalase activity ( Table 3). Irrespective of its presence in PGPE, we employed catechin in all our QS experiments as a positive control, since it is a known QS inhibitor ( Vandeputte *et al.,* 2010). Information on its *in vivo* efficacy has been provided in respective figure legends ( Figure 1D, Figure 2F, Figure 5D).

***Pure compounds against P. aeruginosa.*** With respect to docking score against lasR receptor of *P. aeruginosa*, catechin, querectin and genistein exhibited highest affinity towards this receptor; whereas punicalagin, punicalin, rutin, and pedunculagin exhibited maximum affinity for the pqsR receptor of this bacterium. Preliminary *in vitro* experiments revealed cinnamic acid, chlorogenic acid, and catechin to exert their maximum effect on *P. aeruginosa* QS-regulated pigment production at different concentrations. Data for the most effective concentration for each of these three compounds is presented in Figure 7A–C. Production of both pigments was affected the most by chlorogenic acid, and this compound was also able to make *P. aeruginosa* more susceptible to cephalexin ( Figure 7E); however, it could not alter this bacterium’s susceptibility to ofloxacin and gentamicin. With respect to antibiotic susceptibility result obtained with cinnamic acid ( Figure 7E) were identical to those with chlorogenic acid. AHL augmentation was able to reverse effect of chlorogenic as well as cinnamic acid on production of both the QS-regulated pigments of *P. aeruginosa* ( Figure 7D), suggesting these compounds to disturb the QS machinery of this pathogen by acting as signal-supply inhibitors. Both these compounds were able to negate haemolytic ability of *P. aeruginosa* notably (19.11%; *p* =0.04 and 35.29%; *p=* 8.31363E-05 reduction respectively by cinnamic acid and chlorogenic acid), whereas catalase activity was enhanced to a marginal (1.47%) extent only by chlorogenic acid. *In vivo* efficacy of chlorogenic acid was found to be better than that of cinnamic acid ( Figure 7F).

Catechin could reduce production of pyocyanin and pyoverdine both, in *P. aeruginosa*, without affecting its growth ( Figure 7C). However, there was not much difference in the effects exerted by different concentrations of catechin. For example, the effect on pyoverdine production exerted by all concentrations in the range 10–100 μg/ml was statistically same; and so was the case for the effect exerted by catechin on pyocyanin production in the concentration range of 100–200 μg/ml. Catalase and haemolytic activity of *P. aeruginosa* were both negatively affected by catechin ( Table 3). Catechin seemed to act as a signal-response inhibitor against *P. aeruginosa* ( Figure 7D).

**Figure 8.**
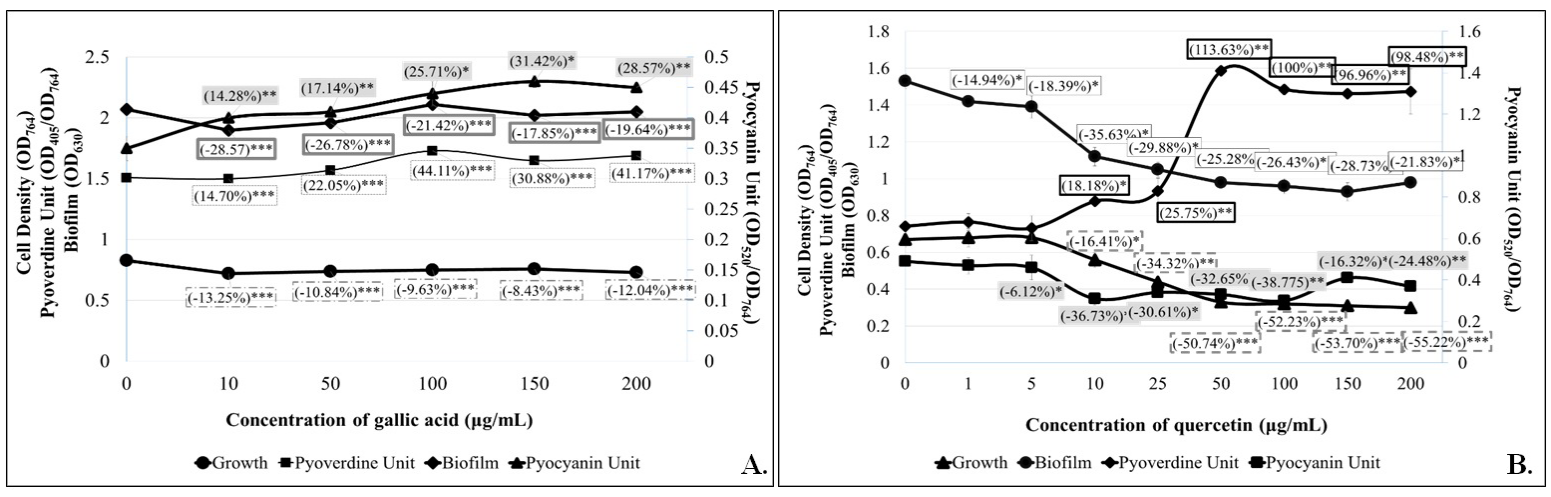
Effect of GA and quercetin on various traits of *P. aeruginosa, in vitro*. ‘Control’ in this figure is the vehicle control (0.5%v/v DMSO), which did not exert any effect on growth and pigment production of *P. aeruginosa*; Bacterial growth was measured as OD _764_; OD of pyoverdine was measured at 405 nm, and Pyoverdine Unit was calculated as the ratio OD _405_/OD _764_ (an indication of pyoverdine production per unit of growth), Pyocyanin Unit was calculated as the ratio OD _520_/OD _764_ (an indication of pyocyanin production per unit of growth); Crystal violet assay was performed to measure biofilm formation, and biofilm eradication, followed by the measurement of OD at 580 nm. ( **A**) Effect of GA on growth and QS-regulated pigment production in *P. aeruginosa*. ( **B**) Effect of quercetin on growth and QS-regulated pigment production in *P. aeruginosa*. * *p*<0.05, ** *p*<0.01, *** *p*<0.001; AS, antibiotic susceptibility; QS, quorum sensing; GA, gallic acid.

GA and quercetin were tested against *P. aeruginosa* at 10–200 μg/ml. Both these compounds were able to inhibit the bacterial growth, and this growth inhibitory effect was more profound in case of quercetin. Production of both the QS-regulated pigments in *P. aeruginosa* was enhanced by GA. Quercetin had a negative effect on pyocyanin production, whereas pyoverdine production was enhanced by it from 10 μg/ml onwards. Catalase activity remained unaffected in GA-treated *P. aeruginosa* culture, whereas it was affected to a minor extent in quercetin-treated culture. Both of these plant compounds were able to reduce biofilm-forming capacity of *P. aeruginosa* notably ( Figure 8A, B).

**Figure 9.**
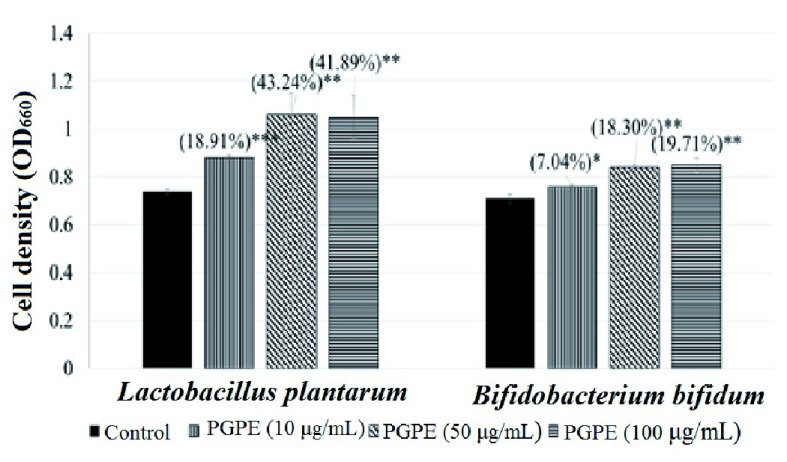
Growth promoting effect of *Punica granatum* peel extract on probiotic strains. Bacterial growth was measured as OD _660_. * *p*<0.05, ** *p*<0.01, *** *p*<0.001. PGPE: *Punica granatum* peel extract.

***Growth promoting effect of PGPE on probiotic strains.*** PGPE was able to enhance growth of *L. plantarum* and *B. bifidum* at 10–50 μg/ml. Higher concentration (100 μg/ml) did not seem to promote their growth further ( Figure 9).

## Discussion

Our *in vitro* and *in vivo* experiments indicated hydroalcoholic extract of *P. granatum* peel to possess appreciable antipathogenic activity against both the test bacteria. Antimicrobial activity of pomegranate extracts against bacteria, fungi, and plasmodium has previously been reported by many researchers ( Rahmani *et al.,* 2017). Other researchers have reported *P. granatum* extracts to possess antimicrobial and/or anti-QS effect. However almost all of them have shown the effective concentrations to be much higher than what we report for PGPE in the present study. Ethanolic extracts of pomegranate were shown to inhibit methicillin-resistant *S. aureus* isolates at 200–400 μg/ml ( Voravuthikunchai & Kitpipit, 2005). A high tannin pomegranate extract was reported to be effective against MRSA at 1–5 mg/ml (Su *et al.,* 2012). *Helicobacter pyroli* isolates were shown to be inhibited by pomegranate extracts at 50–100 μg disc ^-1^. Pomegranate peel extract was reported to inhibit *Salmonella typhimurium* and *Salmonella aureus* on meat surfaces, at 250 μg/ml ( Tayel *et al.,* 2012). Effective concentrations reported in our study are also lower than the quorum-modulatory concentrations reported by Yang *et al.* (2016). They reported tannin-rich fraction from pomegranate rind (TFPR) caused a reduction (~50-70%) in *C. violaceum* pigmentation at 0.312–0.625 mg/ml, whereas comparable pigment inhibition was achieved by our extract at 50–150 μg/ml ( Figure 1A). Oh *et al.* (2015) reported pomegranate peel extract to inhibit bacterial growth at 3–12 mg/ml. Our extract could inhibit violacein production by 78.26% at 10 times lesser concentration (200 μg/ml; Figure 1A) than the concentration (2 mg/ml) they reported for their extract to achieve a comparable (78.5%) violacein inhibition in *C. violaceum*. Inhibition of biofilm formation of human pathogens by *P. granatum* extract at <40 μg/ml reported by Bakkiyaraj *et al.* (2013) matches with our results of the present study ( Figure 2E). Nuamsetti *et al.* (2012) reported pomegranate extracts to exert antibacterial activity against four different bacteria with MIC values reaching 103.6-207 mg/ml.

Much evidence for antimicrobial action of pomegranate extracts has come from *in vitro* assays, whose confirmation through *in vivo* assays is necessary ( Howell & D’Souza, 2013). Dazal *et al.* (2015) reported ethanolic extract of *P. granatum* pericarp to inhibit QS in *P. aeruginosa* at 219.78–2197.80 μg/ml; whereas our extract could modulate production of both QS-regulated pigments from 5 μg/ml onwards *in vitro*; notable *in vivo* efficacy was observed at 0.5 μg/ml onwards. An ethanolic extract of pomegranate exhibited MICs of 0.49–1.95 mg/ml and MBCs of 1.95–3.91 mg/ml against *E. coli* O157:H7. Compared to these relatively higher effective concentrations, PGPE reported in the present study exhibited notable (>50% worm survival, as against 12.5–35% in control wells) *in vivo* efficacy at lower (0.5–5 μg/ml) concentrations ( Figure 1D and Figure 2F).

Higher anti-infective efficacy shown by our extract reported in this study may be attributed to the extraction method employed by us i.e. MAE. This method is known to be capable of fast extraction of phenolics Proestos & Komaitis (2008), and also suitable for heat-labile phytoconstituents ( Gupta *et al.,* 2012). The fact that same extract when prepared using different extraction methods, can vary with respect to its efficacy, has previously also been emphasized by us ( Kothari *et al.,* 2012).

This study has found PGPE to be an effective quorum modulator against two gram-negative bacterial pathogens. An ideal quorum-modulator is expected to have no or minimal effect on bacterial growth, and thus to exert lesser selection pressure on the target pathogenic population. PGPE reported in this study was observed to exert a purely quorum-modulatory effect against *C. violaceum* till 10 μg/ml, and *P. aeruginosa* till 2.5 μg/ml. It is generally expected that owing to lesser effect on bacterial growth, bacterial populations are likely to develop resistance to quorum-targeting antimicrobials at a slower pace. Further, in the case of plant extracts, there is always a possibility of multiple phytocompounds being simultaneously responsible for the observed antipathogenic effect, and they may be targeting multiple cellular components in the target bacteria, hence the susceptible pathogen population may find it difficult to develop resistance against them. Precisely this was observed in our study with *P. aeruginosa*, wherein even repeated exposure of this bacterium to PGPE did not induce resistance, and even the PGPE-habituated *P. aeruginosa* remained susceptible to this extract, *in vitro* ( Figure 2G) as well as *in vivo* ( Figure 2F).

One of the frequently observed problem with conventional antibiotic treatments is that besides killing the target pathogen, they simultaneously disturb the normal human microbiota, which may lead to the condition of dysbiosis. Hence, it is desirable from an anti-infective preparation that it should not have any negative effect on the normal microbiota organisms. This study has found PGPE to promote growth of two bacteria ( *L. plantarum* and *B. bifidum*), which are part of normal human gut biota ( Figure 9). From these results, PGPE can be said to have appreciable prebiotic potential. Li *et al.* (2015) had also indicated pomegranate whole fruit extract to work as a potential prebiotic, with beneficial effect on *Bifidiobacterium* and *Lactobacillus*. Pomegranate extracts can be expected to exert meaningful prebiotic and anti-infective effect *in vivo* in human body *,* as they are rich in ellagitanins, which are hydrolysed in the gut to ellagic acid, which further is metabolized by the colon microbiota to form urolithin A and urolithin B. These urolothins at micromolar concentrations were indicated to be potent *in vivo* QS-inhibitors by Giménez-Bastida *et al.* (2012). Prebiotic preparations with bifidogenic property, besides improving gastrointestinal function, can also find applications in management of conditions like depression and psychological distress ( Messaoudi *et al.,* 2011). Overall GI tract benefits expected from *P. granatum* extracts, as indicated in ethnomedicinal records ( Colombo *et al.,* 2013; Hollebeeck *et al.,* 2012) can be in part due to its prebiotic potential, which makes use of pomegranate in management of inflammatory bowel disease appear relevant.

Our results regarding effect of PGPE on haemolytic activity of both the test bacteria, and its effect on *P. aeruginosa* susceptibility to human serum also indicate the high probability of this extract to be of therapeutic relevance. Haemolysis has been considered as an important virulence factor, and infection by haemolytic bacteria may lead to severe anaemia ( Orf & Cunnington, 2015). By reducing the haemolytic activity of the pathogens, PGPE-like extracts can significantly reduce iron-availability for the pathogens, as lysis of red blood cells is one of the effective strategies for many pathogens to ensure sufficient supply of iron, necessary for their survival inside host. Enhanced susceptibility of *P. aeruginosa*, under influence of PGPE, to lysis by human serum is also of clinical significance, as this can aid the clearance of pathogenic bacteria by human body, while facing bacteremia. This may represent an additional possible mechanism by which PGPE may confer *in vivo* protection against bacterial infection.

Any quorum-inhibitory substance may act either by targeting the signal-synthesis machinery, sequestering the already synthesized signals, or by interfering with the signal-reception ( Zhang & Li, 2016). Though different phytocompounds of a given crude extract may have different mode of action, as a whole extract PGPE was found to act as a signal-response inhibitor against *C. violaceum* ( Figure 1B) and *P. aeruginosa* ( Figure 2B). Among the pure compounds tested in this study, except GA, remaining four compounds seemed to act as a signal-response inhibitor against *C. violaceum*. Notably, cinnamic acid and chlorogenic acid were found to behave as signal-supply inhibitors against *P. aeruginosa*, and as signal-response inhibitor against *C. violaceum*. This suggests that same plant compound may act differently against different bacteria. Here this fact becomes even more interesting, given both *C. violaceum* and *P. aeruginosa* are gram-negative bacteria, and a heavy overlap is suggested to be there among the QS machinery of different members of gram-negative bacterial group ( Papenfort & Bassler, 2016). Since PGPE contains a mixture of signal-supply and -response inhibitors, it can be difficult for the susceptible bacteria to develop simultaneous resistance to both mechanisms (i.e. against the whole extract). Compared to this, it may be easier for the bacteria to develop resistance against single-molecule antibiotics with bactericidal action.

At 0.5–1 μg/ml PGPE showed *in vitro* effect neither on growth, nor on pigment production in any of the test bacteria. In case of *C. violaceum*, any *in vitro* effect was not observed till 2.5 μg/ml. However, all these concentrations that were ‘not effective’ *in vitro* were able to significantly reduce virulence of these bacterial pathogens towards *C. elegans* ( Figure 1D and Figure 2F). This fact highlights the necessity of assaying any test extract at broadest possible concentration range *in vivo*, including at least one level below the lowest effective *in vitro* concentration. Usually *in vitro* screens are performed at a broader concentration range to identify the most effective concentration(s), and *in vivo* assays are conducted at this narrowed concentration range; though logical, this approach carries an inherent risk of missing out some concentrations meaningful *in vivo*. A simple explanation which can be proposed for this apparent mismatch between *in vitro* and *in vivo* results is that, *in vitro* we look for effect of test extract on a limited number of parameters (e.g. only two parameters, cell density and pigment production, in current study), whereas *in vivo* assays provide the test extract an opportunity to interact with multiple targets in host as well as pathogen. We may not be able to study all these targets, but the overall effect can be detected in terms of survival benefit of the model host. Even the magnitude of difference in *in vitro* effects of different concentrations, may not always match with their *in vivo* effect. For example, *in vitro* pyoverdine by *P. aeruginosa* was overproduced 2.51-fold more at 50 μg/ml that that at 25 μg/ml PGPE; but there was no significant difference in the *in vivo* efficacy of these two concentrations. On the same line, *in vitro* effect of PGPE at 25–100 μg/ml on *C. violaceum* pigment production was 42–56%, whereas *in vivo* effect of these concentrations fell within a narrower range of 75–80% (i.e. a 5% *in vivo* difference for 14% *in vitro* difference in efficacy values).

Results of this study also suggest that while screening natural/synthetic compounds for their possible effect on bacterial QS, what we should observe for is ‘quorum modulation’ and not just ‘quorum inhibition’. This is particularly evident from the effect of PGPE on pyoverdine production in *P. aeruginosa*, wherein this virulence factor production is not inhibited by PGPE, but promoted to quite notable extent, and this pyoverdine-enhancing (modulatory) effect of PGPE does not prevent it from being effective *in vivo*. Similar enhancement in pigment production by *Serratia marcescens* challenged with an anti-infective quorum-modulatory polyherbal *Panchvalkal* formulation was reported by us earlier ( Patel *et al.,* 2017), and the formulation was effective *in vivo*.

While investigating the effect of PGPE and/or its constituent phytocompounds against the two gram-negative bacteria in this study, the dose-response relationship was not always found to be linear. Since dose-response relationships can be described by various models ( Calabrese, 2004), we made an effort to see, which model fits best to results obtained in this study. For example, effect of PGPE on *C. violaceum* growth did not follow a linear pattern. Concentrations till 2.5 μg/ml affected neither cell density nor pigment production ( Figure 1A); this phenomenon can be said to fit in the threshold model, wherein the biological effect is not observed until the threshold concentration is crossed. This concept of threshold model also seems applicable while describing effect of PGPE on *P. aeruginosa* cell density and pigment production, as none of the parameters seem to be affected until the threshold concentration of 1 μg/ml was not crossed ( Figure 2A). At higher concentrations, the effect on pyoverdine production seemed to somewhat follow an inverted U-shaped hormetic model. Whether such a pattern would have continued at still higher concentrations, cannot be predicted without actually performing experiment at those concentrations. Similar analysis with respect to pure compounds revealed that effect of GA on *C. violaceum* cell density and pigment production ( Figure 6A) can be said to follow the linear non-threshold model, within the tested concentration range. The term ‘hormesis’ is used in general to describe a biphasic dose response ( Mattson, 2008), and this concept can be relevant while describing ‘adaptive stress response’ in bacteria challenged with some antimicrobial/quorum modulatory agent. Hormetic response can be said to occur (or not to occur) within the concentration range actually tested during a particular experiment, but it need not to be extrapolated into the realm of uncertain i.e. concentrations not really tried, on either side of the test concentration range.

Overall, the whole extract seemed to be more efficacious than any of the pure compounds tested. For example, at 10 μg/ml, PGPE affected pyoverdine production in *P. aeruginosa* much more than either GA or quercetin. Such synergistic action of the pomegranate constituents, apparently being superior to that of its individual constituents, has been indicated in literature ( Jurenka, 2008). Among the many constituents of PGPE, GA in this study was found to act as a signal-supply inhibitor against *C. violaceum*, whereas all other compounds as well as the whole extract were found to act as signal-response inhibitor against this bacterium. There may be few other unknown compounds present in this extract, which may either inhibit AHL synthesis, sequester synthesized AHLs, or bind to the LuxR component. It is likely to be difficult for the bacteria to develop complete resistance against any such poly-component preparation, as different compounds will target different components of the bacterial QS machinery. Anand *et al.* (2013) has also indicated the combination of LuxR non-competitive inhibitors and LuxI inhibitors, as a robust therapeutic strategy to act multiplicatively across a broad parameter range.

*In silico* docking exercise indicated both, quercetin and, catechin to have affinity for CviR more than its natural ligand C6-HSL ( Table 2), i.e. a high probability of them being capable of acting as signal-response inhibitor. *In vitro* experiments validated this possibility ( Figure 6D, F). Though the docking scores of both these phytochemicals against CviR were identical, *in vitro* violacein production was inhibited bit more by quercetin. Gallic acid, which showed second lowest affinity (-5.9 kcal/mol) for CviR among all docked phytocompounds, was also not able to act as signal-response inhibitor, instead it acted as signal-supply inhibitor against *C. violaceum*, and its docking score against the signal synthesizing enzyme AHL synthase was also better i.e. (-7.9 kcal/mol). Cinnamic acid displayed lowest *in silico* binding affinity against *P. aeruginosa* receptor protein PqsR ( Table 2), and this corroborates well with the fact revealed from *in vitro* experiment that it acts as a signal-supply inhibitor ( Figure 7D). Though the binding affinity of catechin and quercetin both, against *P. aeruginosa* protein LasR was identical as per docking score, indicating a high probability of them acting as a signal-response inhibitor with respect to pyoverdine, whose production is controlled by the las system, and *in vitro* experiments did find catechin to act as a signal-response inhibitor ( Figure 7D); pyoverdine production was affected much more by quercetin than by catechin. If we focus on docking scores of only those five compounds, with which *in vitro* experiments were also conducted in this study, against both the *P. aeruginosa* receptors, quercetin gives an impression of being more effective than GA, and *in vitro* experiments also show quercetin to have more effect on pigment production by this bacterium, than GA ( Figure 8A and 8B). Similarly docking scores of catechin against both *P. aeruginosa* receptor proteins were better than that of cinnamic acid ( Table 2), and *in vivo* assay also found catechin to be more efficacious than cinnamic acid ( Figure 7F). Overall there seems to be an empirical match between the *in silico* and wet-lab experiments.

This study has found PGPE to be an effective quorum-modulator against two different antibiotic resistant strains of gram-negative bacteria. In general, it is more challenging to find antimicrobials effective against gram-negative bacteria, than against gram-positive bacteria, mainly for the reason that the former possess an additional barrier in form of outer membrane, posing extra-challenge to the intracellular entry of any antimicrobial. Besides affecting QS-regulated pigment production in the test bacteria, this extract could also effectively modulate other traits, including catalase activity, haemolytic activity, biofilm formation, susceptibility of bacteria to antibiotics and to lysis by human-serum factors. Though researchers have reported the antimicrobial potential of *P. granatum* extracts ( Al-Zoreky *et al.,* 2009; Choi *et al.,* 2011; Prashanth *et al.,* 2001) or nanoparticles derived from it ( Devanesan *et al.,* 2018), reports describing *in vivo* anti-infective efficacy of PGPE with focus on its quorum modulatory potential are still warranted. This extract was not found to inhibit growth of test bacteria too heavily, at effective quorum-modulatory concentrations, and this is what is expected from a ‘good’ quorum-modulator (i.e. having no or minimal effect on growth of the test bacterium, and exerting no great selection pressure on the pathogen population).

Further studies regarding elucidation of the molecular mechanism associated with such appreciable anti-infective activity of PGPE is warranted, as that will give us a better insight into its mode of action. This study also highlights the importance of using different phytocompounds in combination (e.g. as a whole extract; concept of synergy), rather than always focusing on the single-active-molecule approach. Results reported here can be said to be supportive of the concept of polyherbalism ( Parasuraman *et al.,* 2014) widely employed by the practitioners of complementary and alternative medicine.

## Data availability

### Underlying data

All raw data containing the underlying data behind each figure and table are available on figshare. DOI: https://doi.org/10.6084/m9.figshare.7471979 ( Joshi *et al.,* 2018).

### Extended data

Extended data are available on figshare. DOI: https://doi.org/10.6084/m9.figshare.7471979 ( Joshi *et al.,* 2018).

Figure S1. Antipathogenic effect of Pomella on *P. aeruginosa*.

Figure S2. Quorum-inhibitory effect of cinnamic and chlorogenic acid on violacein production in *C. violaceum* is not reversed following augmentation of the quorum-inhibited culture by AHL

Table S1. Binding affinity of different phytocompounds when docked against LuxR homologue of test pathogens.

Data are available under the terms of the Creative Commons Zero "No rights reserved" data waiver (CC0 1.0 Public domain dedication).
