# Supplementary material for: Anti-infective potential of hydroalcoholic extract of Punica  granatum peel against gram-negative bacterial pathogens
Source: F1000Res. 2019 Apr 8;8:70. Originally published 2019 Jan 18. [Version 2] doi: 10.12688/f1000research.17430.2 (PMC6392158; doi:10.12688/f1000research.17430.2)
Supplement: Peer review history

# Peer review: Reviewer response for version 1

**DOI:** 10.5256/f1000research.19060.r43898

**Published:** 2019-02-25

**Authors:** Shilpa Deshpande Kaistha

**Affiliations:** 1 Department of Microbiology, Chhatrapati Shahu Ji Maharaj University (CSJMU), Kanpur, India

This study provides comprehensive experimental evidence for the activity of Punica granatum peel extract in modulating pathogenicity of *Pseudomonas* *aeruginosa* and quorum sensing pigment producing *Chromobacterium* *violaceum*. The effect of PGPE on various aspect of pathogenicity related to antibiotic sensitivity, biofilm formation, surface hydrophobicity, quorum sensing, hemolysin & catalase production and *in* *vivo* *C. elegans* infection model together make a comprehensive and elegant study of the herbal extract activity. The effect of concentration dependent PGPE treatment on various parameters and subsequent generations of the pathogens make for an interesting read. It is recommended that the article be indexed with some minor improvements as suggested below:

**Introduction** **:**

 At several places, relevant references may be cited.

**Material and Methods:**

1. Pigment production can be separate heading and subheadings for each pigment, to make clear. Growth measurement can be included in the write up.
2. Details of Chloramphenicol enhanced catalase maybe provided.

**Results** **:**

1. The quality of all the figures requires significant improvement. The details of significant values maybe kept in the figures to avoid crowding and enhance understanding. Font size of Figures, for example Figure 2C, needs to be clarified. The standard deviations are not visible in most of the figures.
2. A brief summary of comprehensive results at the end of each section will help the reader grasp the crux of the experiment without having to go through the written text regarding working concentrations of the extracts etc.

**Discussion:**

1. Requires a section wherein the findings are succinctly concluded regarding the findings for *Ps. aeruginosa* and *C. violaceum*.
2. Please include in Discussion why does the PGPE enhance probiotic activity while showing anti-infective activity against the pathogens studied.

I have read this submission. I believe that I have an appropriate level of expertise to confirm that it is of an acceptable scientific standard, however I have significant reservations, as outlined above.

# Peer review: Reviewer response for version 1

**DOI:** 10.5256/f1000research.19060.r43897

**Published:** 2019-02-21

**Authors:** Sisir Nandi

**Affiliations:** 1 Department of Pharmaceutical Chemistry, Global Institute of Pharmaceutical Education and Research, Affiliated to Uttarakhand Technical University, Kashipur, India; 2 Uttarakhand Technical University, Kashipur, India

Chinmayi and co-workers studied hydroalcoholic extract of *P. granatum *peel (PGPE), prepared by microwave assisted extraction method for its quorum-modulatory potential against two different human-pathogenic bacteria viz. *Chromobacterium violaceum *and *Pseudomonas aeruginosa*. This is a very significant work to produce antimicrobials. The following minor comments should be considered before acceptance of this article.

  

1. "Though the modern medicine has almost always focused on search of purified active molecules as therapeutic leads, knowledge of Indian/Chinese/Arabian traditional medicine makes us aware of a variety of plant extracts and inorganic formulations prescribed for management of infections". Cite the references.
2. "One such widely mentioned item in these ancient systems of complementary and alternative medicine, including the Indian system *Ayurved*, is *Punica granatum *L. extracts". Cite the references.
3. "In India, Tunisia, and Guatemala, decoction of driedpeels of *P. granatum *is employed externally as well as internally as an astringent and germicide, and utilized for treating aphthae and diarrhoea". Cite the references.
4. "Klebseiella Pneumonia" Spelling mistake is there. It should be corrected as klebsiella pneumonia.
5. "Incubation temperature and time for *C. violaceum*, *L. plantarum*, and *B. bifidum *was 37°C, and 22–24 h". Cite the references.
6. "Antibiotic susceptibility profile of the bacterial strains used in this study was generated using the antibiotic discs- Dodeca Universal – I, Dodeca G - III – Plus, and Icosa Universal -2 (HiMedia, Mumbai)". Cite the references.
7. "The plant name was checked against http://www.theplantlist.org on April 20, 2018. Collected peels were shade-dried, before being used for extract preparation. Biological activity of the plant fruit even depends on the time and season of productions". This fruit is generally produced in season in the Northern Hemisphere from September to February and in the Southern Hemisphere from March to May. Please comment on this. Authors are being also suggested to carry out the same experiment using the Peels of *P. granatum *were procured from different times. (optional)
8. "Purity of each of the extracted pigment was confirmed by running a UV-vis scan (Agilent Cary 60 UV-visible spectrophotometer). Appearance of single major peak (at the λ _max_x reported in literature) was taken as indication of purity". Cite reference.  
9. Fig 4 is not clear and acceptable in the present form. Please prepare a high resolution figure as per the authors instruction.
10. Why did the author do molecular docking study? It should be clearly mentioned in the methods section along with references ^1^^,^^2^. Docking score is not the ultimatum measurement of potency or binding affinity of the ligand. I suggest giving the figures of mode of ligand-receptor interactions and discussing the crucial interacting amino acids of the protein.
11. "One ethnomedical practice reported from the Paliyar tribe from Tamilnadu in India involves taking internally, dried fruit coat of *P. granatum *after grinding and mixing with water, to treat stomach ache and diarrhoea (Duraipandiyan *et al., *2006)". Correct the typographical error of Tamilnadu.
12. The present version contains many grammatical and typographical errors. Authors are suggested to do these corrections.
13. "Inoculum standardized to 0.5 McFarland turbidity standard was added at 10% v/v, to the media supplemented with required concentration of PGPE, followed by incubation at appropriate temp for each organism". Correct the spelling error of temp.
14. Separate conclusion should be given based on experimental and in-silico results.

I have read this submission. I believe that I have an appropriate level of expertise to confirm that it is of an acceptable scientific standard, however I have significant reservations, as outlined above.

## References

[1] NandiS: Recent Advances in Ligand and Structure Based Screening of Potent Quorum Sensing Inhibitors Against Antibiotic Resistance Induced Bacterial Virulence. *Recent Patents on Biotechnology*.2016;10(2) : 10.2174/1872208310666160728104450 195-216 10.2174/1872208310666160728104450 27468815

[2] NandiSBagchiMC: 3D-QSAR and molecular docking studies of 4-anilinoquinazoline derivatives: a rational approach to anticancer drug design. *Mol Divers*.2010;14(1) : 10.1007/s11030-009-9137-9 27-38 10.1007/s11030-009-9137-9 19330460

# Peer review: Reviewer response for version 1

**DOI:** 10.5256/f1000research.19060.r43899

**Published:** 2019-02-19

**Authors:** Saif Hameed

**Affiliations:** 1 Amity University Haryana, Gurugram, India

In this study, the authors analyze an “Anti-infective potential of hydroalcoholic extract of Punica granatum peel against gram-negative bacterial pathogens” with various experiments QS, biofilm hemolytic activity, catalase activity, surface hydrophobicity. Authors also performed *in-vivo* activity with *C. elegans* model organism and also analyzed with docking tool. While this study is comprehensive and important for better understanding of gram negative bacteria ( *C. violaceum *and *P. aeruginosa*) physiology and pathogenesis, experiments are enough and paper sounds good but requires minor corrections with respect to the overall presentation of the manuscript.

**Comment 1:**  In the introduction, the ESRC reference should be changed as it is from 2014.

**Comment 2**: Brief description about AMR and how bacteria develop these mechanism would be better. 

**Comment 3:** Broth dilution assay: Give the proper reference of MIC determination method and how they defined MIC for example (MIC50, MIC80 or MIC90) or complete at the end point of experiment. Better to put the reference of MIC according to CLSI guidelines.

**Comment 4**: Measurement of bacterial growth and pigment production: better to merge this sentence with above and keep the pigment production in separate heading.

**Comment 5** *: In vivo *assay: last day of the experiment, when plates could be opened, their death was also confirmed by touching them with a straight wire, wherein no movement was taken as confirmation of death. Please mention what metal was used.

**Comment 6**: In Results section:

- Figure 1A: is not clear and difficult to understand. The values shown in boxes or brackets - what are these values defining? Are they necessary to show? If yes, better to show in table form.
- Similarly for Fig 1C.
- Figures 1D: Better to show *C. elegans* survival graph along with their pictures (Fig. 1D 2F, 3B).
- Figure 2: very overcrowded and messy. Is it necessary to show 5 figures A,B,C,D,E,F in a single panel? If there is any figure limitation then show only relevant ones and put rest as supplementary figures.

I have read this submission. I believe that I have an appropriate level of expertise to confirm that it is of an acceptable scientific standard.
